# Untargeted Metabolomic and Phytochemical Profiling of *Berberis crataegina* DC. Plant Parts: Antidiabetic Potential and Cytoprotection in High-Glucose-Exposed SH-SY5Y Cells

**DOI:** 10.3390/ph19091365

**Published:** 2026-08-28

**Authors:** Yiğit Erkmen, Zekiye Ceren Arıtuluk Aydın, Engin Koçak, Hasya Nazlı Gök, Emirhan Nemutlu, Merve Yüzbaşıoğlu Baran

**Affiliations:** 1 Department of Pharmacognosy, Gulhane Faculty of Pharmacy, University of Health Sciences, 06108 Ankara, Türkiye; yigit.erkmen@sbu.edu.tr; 2 Department of Pharmaceutical Botany, Faculty of Pharmacy, Hacettepe University, 06100 Ankara, Türkiye; zceren@hacettepe.edu.tr; 3 Department of Analytical Chemistry, Gulhane Faculty of Pharmacy, University of Health Sciences, 06108 Ankara, Türkiye; engin.kocak@sbu.edu.tr; 4 Department of Pharmacognosy, Faculty of Pharmacy, Gazi University, 06330 Ankara, Türkiye; hasyaekin@gazi.edu.tr; 5 Department of Analytical Chemistry, Faculty of Pharmacy, Hacettepe University, 06100 Ankara, Türkiye; enemutlu@hacettepe.edu.tr

**Keywords:** alpha-glucosidase, *Berberis* spp., untargeted metabolomics, antioxidant, cytoprotection, polyphenols

## Abstract

**Background/Objectives:** *Berberis* species are recognized for their ethnomedicinal importance and phytochemical richness associated with antidiabetic activity. Although *B. crataegina* is widely distributed in the Turkish flora and has a history of traditional use, its phytochemical properties and biological activities remain less extensively investigated than those of other *Berberis* species. This study aimed to comparatively evaluate the phytochemical profiles, in vitro antidiabetic potential, and cytoprotective effects of extracts prepared from different parts of *B. crataegina* in a high-glucose-induced SH-SY5Y cell-based model of diabetic neuropathy. **Methods:** Crude hydroethanolic extracts (70% ethanol) were prepared from the leaves, flowers, shoots, roots, and fresh fruits of *B. crataegina* collected from Kızılcahamam, Ankara, Türkiye. The total phenolic and flavonoid contents, antioxidant capacities and α-glucosidase inhibitory activities of leaf, flower, shoot, root, and fruit extracts were determined. The cytoprotective effects of the crude extracts were evaluated in a high-glucose-induced SH-SY5Y cell model. The metabolomic profiles of the extracts were comparatively analyzed using liquid chromatography–quadrupole time-of-flight mass spectrometry (LC-QTOF-MS). The most active fruit extract was fractionated by reversed-phase vacuum liquid chromatography (RP-VLC), and the phytochemical contents and α-glucosidase inhibitory activities of the fractions were evaluated. The major phenolic compounds in the most active fruit extract were quantitatively determined using high-performance liquid chromatography–diode array detection (HPLC-DAD). The relationships between metabolites and biological activities were investigated through correlation analysis. **Results:** The fruit extract was distinguished by its α-glucosidase inhibitory activity, total phenolic content, antioxidant capacity, and cytoprotective effect in the high-glucose-induced SH-SY5Y cell model. It showed no marked cytotoxicity at concentrations ranging from 25 to 400 µg/mL and, at 25 µg/mL, attenuated the high-glucose-induced reduction in cell viability, restoring viability to a level close to that of the normal control. Untargeted metabolomic analysis identified 191 metabolites detected in at least two crude extracts, while the metabolites detected exclusively in the fruit extract were predominantly flavonoids and flavonoid glycosides. Fractionation showed that α-glucosidase inhibitory activity was mainly concentrated in the medium-polarity fractions; however, none of the fractions reached the activity level of the crude extract. Chlorogenic acid, rutin, caffeic acid, protocatechuic acid, quercetin, and quercetin-3-O-glucoside were detected and quantified in the fruit extract by HPLC-DAD. Correlation analysis indicated that the observed biological activities may be associated with phenolic acids, flavonoids, and other polyphenolic metabolites. **Conclusions:** The fruit extract of *B. crataegina* showed promising α-glucosidase inhibitory activity and cytoprotective effects in the high-glucose-induced SH-SY5Y cell model. The findings suggest that these effects may arise from the combined contribution of multiple constituents within a polyphenolic matrix rather than from a single compound; however, synergistic interactions were not experimentally demonstrated. These results support further evaluation of the fruit extract through mechanistic, in vivo, and standardization studies.

## 1. Introduction

Diabetes mellitus is a chronic metabolic disorder characterized by persistent hyperglycemia and associated with severe long-term complications, including cardiovascular diseases, diabetic neuropathy, and diabetic foot syndrome [1,2,3]. The global prevalence of diabetes continues to rise, emphasizing the urgent need for effective preventive and therapeutic strategies [4]. Among all complications of diabetes, a group of clinical syndromes caused by damage to the peripheral and autonomic nervous systems is by far the most common. With the increasing prevalence of diabetes worldwide, diabetic neuropathy (DN) has emerged as a global health problem [5].

Among current therapeutic approaches for the management of postprandial hyperglycemia, inhibition of carbohydrate-hydrolyzing enzymes such as α-glucosidase has attracted considerable attention. By delaying intestinal glucose absorption, *α*-glucosidase inhibitors reduce postprandial glucose excursions and contribute to improved glycemic control [6,7]. However, the use of synthetic α-glucosidase inhibitors is frequently associated with gastrointestinal adverse effects, increasing interest in natural alternatives derived from medicinal plants [8,9].

One of the most widely used models for investigating diabetic neuropathy is the SH-SY5Y neuroblastoma cell line. SH-SY5Y cells, a thrice-cloned subline of the SK-N-SH cell line, show responses under high-glucose conditions similar to those observed in dorsal root ganglion neurons and Schwann cells. Therefore, this cell line is considered a suitable in vitro model for evaluating alterations in signal transduction, nitric oxide production, and Na^+^/K^+^ pump activity [10].

Natural products have long been recognized as valuable sources of bioactive metabolites with therapeutic potential against metabolic disorders [7]. In particular, edible medicinal plants represent an important interface between traditional medicine and functional foods, providing structurally diverse secondary metabolites [7,11]. These metabolites, including phenolic acids, flavonoids, alkaloids, terpenoids, and saponins, exhibit antioxidant, anti-inflammatory, and carbohydrate-hydrolyzing enzyme inhibitory activities that are relevant to diabetes management [11,12]. Owing to these biological properties, plant-derived phytochemicals have attracted considerable interest as promising candidates for the development of nutraceuticals and phytopharmaceuticals targeting diabetes and its complications [7,13].

Among medicinal plants traditionally used for diabetes, species of the genus *Berberis* have attracted particular interest because of their rich phytochemical composition and broad spectrum of pharmacological activities. These pharmacological properties have largely been attributed to their diverse secondary metabolites, particularly isoquinoline alkaloids, including berberine, together with various phenolic compounds. Previous studies have reported antioxidant, anti-inflammatory, antimicrobial, hepatoprotective, cytotoxic, and antidiabetic activities for different *Berberis* species. Among these, the antidiabetic and metabolic regulatory properties have received particular attention. Extracts and bioactive constituents of *Berberis* species have been investigated in experimental and clinical studies, with findings indicating potential beneficial effects against diabetes and related metabolic disorders [14,15].

*Berberis crataegina* DC., a species widely distributed in Türkiye, has attracted attention owing to its traditional use, especially in the management of diabetes. Ethnobotanical records indicate that different parts of the plant—including the root, root bark, stem, stem bark, shoots, leaves, flowers, fruits, and branches—have been utilized in folk medicine for various ailments. Among these, the root, stem bark, leaves, fruits, and flowers are most frequently reported for their antidiabetic use across different regions. These plant parts are generally prepared as decoctions or infusions and administered orally; notably, the leaves and fruits are also consumed raw or fresh for diabetes-related purposes. Such traditional practices have been documented in Kayseri, Denizli, Tunceli, Kahramanmaraş, Muğla, Malatya, Karaman, Erzincan, Mersin, Iğdır, and Sivas, highlighting the widespread ethnomedicinal relevance of *B. crataegina* in diabetes management [16,17,18,19,20,21].

Despite its widespread traditional use, scientific evidence concerning *B. crataegina* remains fragmented. Previous studies have investigated individual plant parts, primarily the roots and fruits, and have focused on selected biological activities [22,23,24,25,26,27]. However, a comprehensive comparative evaluation of the phytochemical profiles and bioactivities of different plant parts, particularly in relation to their antidiabetic potential, is still lacking. Moreover, to the best of our knowledge, the protective effects of *B. crataegina* on neuronal cells under high-glucose conditions have not previously been investigated.

Addressing this gap requires an analytical approach capable of comprehensively characterizing and comparing the chemically diverse metabolites present in different plant parts. Untargeted metabolomic analyses are widely used to reveal the chemical profiles of medicinal plants containing diverse groups of secondary metabolites. However, due to the chemical diversity of metabolites, the compound groups that can be analyzed may vary depending on the analytical platform used, method parameters, and sample preparation steps. Therefore, since a single analytical platform cannot cover all metabolites present in medicinal plants, different techniques, such as nuclear magnetic resonance (NMR) spectroscopy, gas chromatography–mass spectrometry (GC-MS), and liquid chromatography–mass spectrometry (LC-MS), are employed; among these, GC-MS and LC-MS are more commonly preferred in metabolomic studies. Metabolite identification is one of the fundamental steps in metabolomic data analysis, and the databases used can directly affect this process. In GC-MS-based metabolomic studies, the combined evaluation of fragmentation spectra and retention indices provides more reliable identification, whereas in LC-MS-based studies, the diversity of fragmentation patterns makes metabolite identification more complex. In this process, metabolite annotation can be performed using KEGG, Reactome, PubChem, METLIN, HMDB, MassBank, GNPS, ReSpect, GMD, KNApSAcK, LIPID MAPS, PlantCyc, and various NMR databases [28].

In this context, LC-QTOF-MS-based untargeted metabolomics enables detailed profiling of complex phytochemical matrices and facilitates the comparative evaluation of metabolite distributions among different plant parts. When integrated with enzyme inhibition and cell-based bioactivity assays, this approach may help identify metabolite classes associated with the observed antidiabetic and cytoprotective effects.

Therefore, this study aimed to comparatively evaluate the biological potential and phytochemical composition of extracts prepared from the leaves, flowers, shoots, roots, and fruits of *Berberis crataegina* DC. in relation to diabetes and diabetic neuropathy. Accordingly, the α-glucosidase inhibitory effects of the extracts were comparatively determined, and their cytoprotective potential was investigated in a high-glucose-induced SH-SY5Y cell-based diabetic neuropathy model. To support the biological activity findings, the total phenolic and flavonoid contents of the extracts, as well as their antioxidant capacities using 2,2-diphenyl-1-picrylhydrazyl (DPPH) radical-scavenging and cupric reducing antioxidant capacity (CUPRAC) assays, were comparatively evaluated. In addition, the phytochemical profiles of the extracts were comparatively characterized by liquid chromatography–quadrupole time-of-flight mass spectrometry (LC–QTOF–MS)-based untargeted metabolomic analyses, and correlation analyses were performed to identify metabolites potentially associated with biological activities. Finally, the amounts of selected marker phenolic compounds were determined by high-performance liquid chromatography–diode array detection (HPLC–DAD) in the fruit extract, which stood out in terms of biological activity.

## 2. Results

The amounts of powdered plant materials used in the preparation of the main extracts, the weights of the resulting extracts, and the extraction yields for each sample are presented in Table 1. The extraction yield was calculated by dividing the weight of the obtained extract by the amount of powdered plant material used and multiplying the result by 100 (extract weight/plant material weight × 100).

### 2.1. Total Phenolic Content

The total phenolic contents of the main extracts of *B. crataegina* DC. are presented in Table 2. The fruit extract exhibited the highest total phenolic content (267.11 mg GAE/g extract), followed by the shoot extract (189.85 mg GAE/g extract), whereas the root extract showed the lowest value (135.78 mg GAE/g extract).

### 2.2. Total Flavonoid Content

The total flavonoid contents of the main extracts of *Berberis crataegina* DC. are presented in Table 3. The fruit extract exhibited the highest total flavonoid content (46.55 mg QE/g extract).

### 2.3. DPPH Radical Scavenging Antioxidant Activity

**Figure 1:**
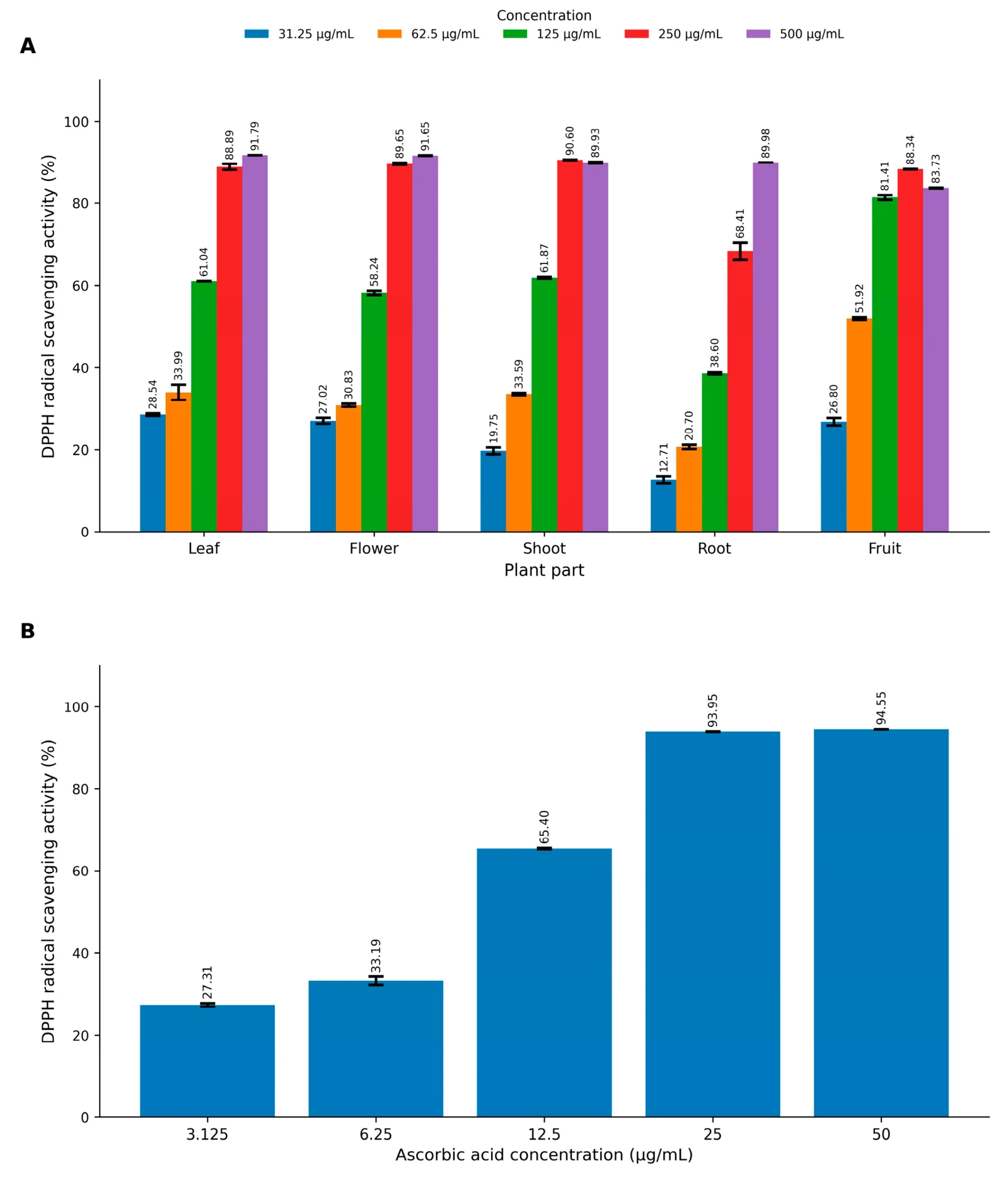
DPPH radical-scavenging activities of the main extracts of *Berberis crataegina* DC. and ascorbic acid. (**A**) Main extracts tested at concentrations of 31.25–250 µg/mL; (**B**) ascorbic acid tested at concentrations of 3.125–25 µg/mL.

The DPPH radical-scavenging activities of the main extracts obtained from the leaves, flowers, shoots, roots, and fruits of *B. crataegina* were evaluated over a concentration range of 31.25–250 µg/mL. Ascorbic acid, used as the positive control, was tested at concentrations ranging from 3.125 to 25 µg/mL (Figure 1). The results were evaluated based on the RC_50_ values, defined as the concentrations required to reduce 50% of the DPPH radicals (Table 4).

The fruit extract exhibited the highest DPPH radical-scavenging activity, with an RC_50_ value of 60.11 µg/mL.

### 2.4. CUPRAC Antioxidant Activity

The shoot extract exhibited the highest antioxidant capacity in the CUPRAC assay, with a value of 243.34 mg GAE/g extract (Table 5).

### 2.5. α-Glucosidase Inhibitory Activity

#### 2.5.1. Crude Extracts

The α-glucosidase inhibitory activities of the crude extracts obtained from the leaves, flowers, shoots, roots, and fruits of *B. crataegina* were evaluated over a final concentration range of 100–1600 µg/mL. The extracts were compared with one another and with acarbose, which was used as the positive control.

Evaluation of the α-glucosidase inhibitory activities of the crude extracts obtained from different parts of *B. crataegina* showed that the leaf and flower extracts did not exhibit significant activity at any of the final concentrations tested. The root extract produced only low, concentration-independent inhibition of approximately 15% at final concentrations of 100 and 200 µg/mL, with no marked effect observed at the other concentrations.

The α-glucosidase inhibitory activities of the shoot and fruit extracts of *Berberis crataegina* DC., together with acarbose as the positive control, at concentrations ranging from 100 to 1600 µg/mL are presented in Table 6. The shoot extract exhibited low α-glucosidase inhibitory activity over the final concentration range of 200–1600 µg/mL. In contrast, the fruit extract showed a high level of inhibition (IC_50_ = 140.45 µg/mL), and its activity was considered comparable to that of acarbose used as the positive control (IC_50_ = 504.11 µg/mL).

#### 2.5.2. Fractions of Fruit Extracts

The α-glucosidase inhibitory activities of fractions (Fr.) A–G obtained from the most active fruit extract of *Berberis crataegina* DC. by reversed-phase vacuum liquid chromatography (RP-VLC), at concentrations ranging from 100 to 1600 µg/mL, are presented in Table 7. Fr. A exhibited the strongest inhibitory activity, with inhibition values ranging from 50.81 ± 1.81% to 69.00 ± 0.04% between 100 and 400 µg/mL. Fr. F and Fr. G also showed notable activity at lower concentrations; Fr. F produced 48.07 ± 1.06% inhibition at 100 µg/mL, whereas Fr. G exhibited 51.02 ± 0.74%–54.15 ± 1.64% inhibition between 100 and 400 µg/mL. Fr. E reached a maximum inhibition of 43.33 ± 1.25% at 400 µg/mL, while no detectable inhibitory activity was observed for Fr. D at any tested concentration.

### 2.6. Cell Culture Findings on the Protective Effects Against Diabetic Neuropathy

#### 2.6.1. In Vitro Neurotoxicity and the Safe Concentration Range in SH-SY5Y Neuroblastoma Cells

The cytotoxicity assay performed to determine the safe concentration ranges for subsequent experiments showed that the fruit extract maintained cell viability between 87.24% and 102.59% over the concentration range of 25–400 µg/mL, indicating no marked cytotoxicity. The shoot and leaf extracts also maintained cell viability above 80% at most tested concentrations. In contrast, the root extract exhibited concentration-dependent cytotoxicity, with cell viability decreasing from 71.40% at 25 µg/mL to 4.40% at 400 µg/mL (Table 8). Accordingly, the concentration ranges selected for the in vitro DN model were 25–400 µg/mL for the fruit, shoot, and leaf extracts and 1.56–25 µg/mL for the root extract.

#### 2.6.2. SH-SY5Y Cell Viability Under High-Glucose Conditions

Following exposure to 50 mM glucose, SH-SY5Y cell viability decreased to 83.91% relative to the normal control group (100%), confirming the successful establishment of the hyperglycemic injury model. In the DN model used to evaluate the main extracts (Table 9), the fruit extract, which had also shown activity in the antidiabetic assays, increased cell viability by approximately 22% at 25 µg/mL compared with the injured control and restored viability to a level close to that of the normal control (approximately 103%).

### 2.7. Quantitative HPLC-DAD Profile of the Fruit Extract

Protocatechuic acid, chlorogenic acid, caffeic acid, rutin, quercetin-3-O-glucoside, and quercetin were detected in the most active fruit extract among the investigated phenolic and flavonoid compounds by HPLC-DAD analysis. The method validation parameters, including linearity, LOD, LOQ, precision, and recovery, are presented in Table 10. All calibration curves exhibited excellent linearity (R^2^ ≥ 0.9998) over their respective concentration ranges. LOD and LOQ values ranged from 0.0002 to 0.0167 ppm and 0.0006 to 0.0507 ppm, respectively, indicating high sensitivity of the method. Intra-day precision (RSD%) ranged from 0.15% to 1.69%, while intermediate (inter-day) precision RSD% values ranged from 1.91% to 7.67% across the six compounds, confirming satisfactory reproducibility of the method. Recovery values, determined at three spiked concentration levels for each compound, ranged from 72.98% to 124.72% (Table 10).

Among these compounds, chlorogenic acid was the most abundant, followed by rutin and caffeic acid, whereas protocatechuic acid, quercetin, and quercetin-3-O-glucoside were detected at lower levels.

**Figure 2:**
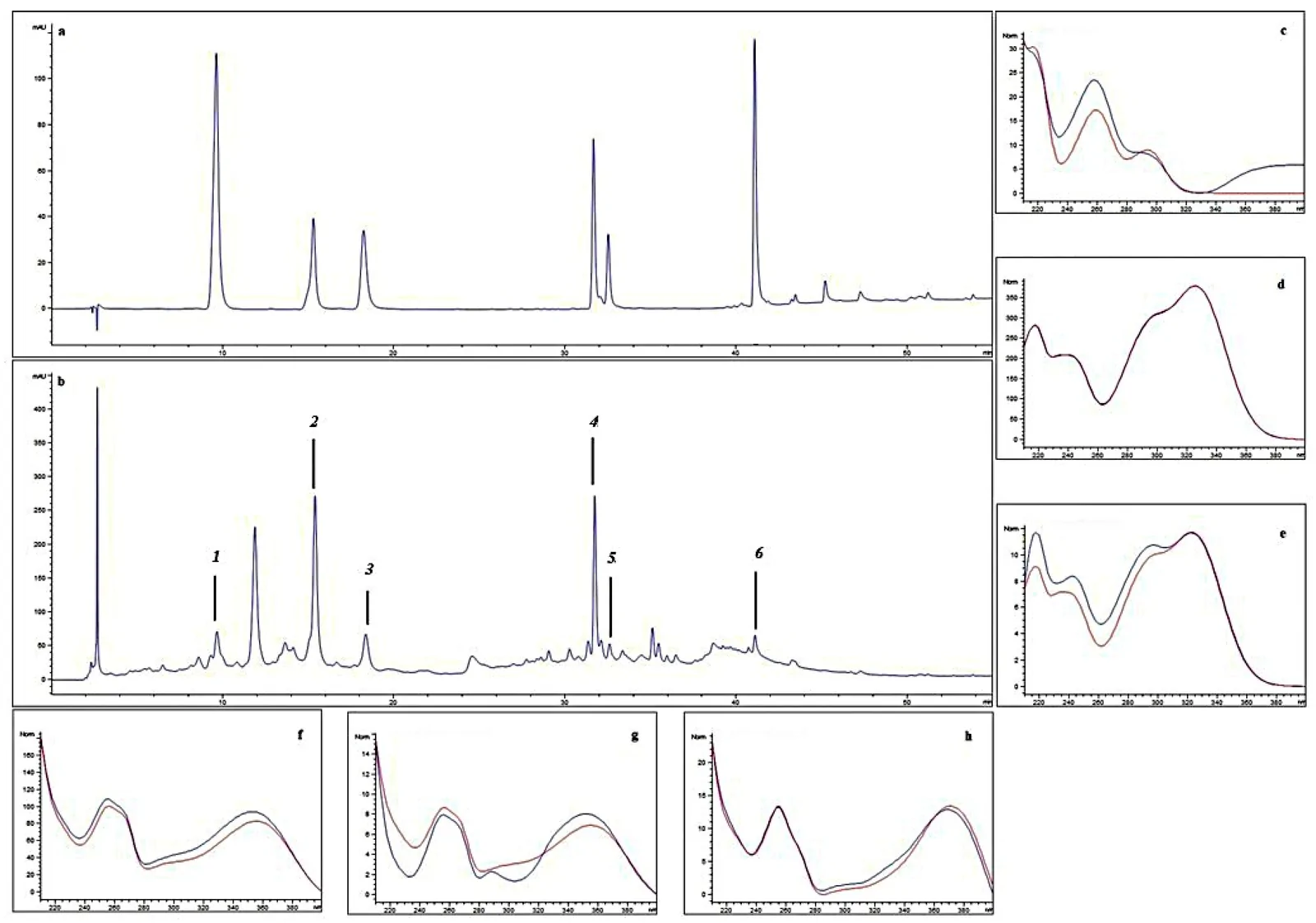
HPLC-DAD chromatograms of the fruit extract recorded at 260 nm and UV spectral overlays with reference standards. (**a**) HPLC-DAD chromatogram of the standard mixture. (**b**) HPLC-DAD chromatogram of the fruit extract: 1, protocatechuic acid; 2, chlorogenic acid; 3, caffeic acid; 4, rutin; 5, quercetin-3-O-glucoside; 6, quercetin. (**c**–**h**) UV spectral overlays of the standard and extract peaks corresponding to protocatechuic acid, chlorogenic acid, caffeic acid, rutin, quercetin-3-O-glucoside, and quercetin, respectively.

The HPLC-DAD chromatograms of the standard mixture and the fruit extract recorded at 260 nm, together with the UV spectral overlays of the detected compounds and their corresponding reference standards, are shown in Figure 2. The spectral overlaps supported the identification of protocatechuic acid, chlorogenic acid, caffeic acid, rutin, quercetin-3-O-glucoside, and quercetin in the fruit extract.

Quantitative analysis was performed using calibration curves prepared with the corresponding reference standards. Based on the calibration data, the contents of chlorogenic acid, rutin, caffeic acid, protocatechuic acid, quercetin, and quercetin-3-O-glucoside in the fruit extract were determined as 5.8003 ± 0.1484, 5.0736 ± 0.0576, 1.2039 ± 0.0026, 0.5395 ± 0.0124, 0.4554 ± 0.0051, and 0.3082 ± 0.0188 mg/g extract, respectively (Table 11).

These findings indicate that the fruit extract has a phenolic/flavonoid profile particularly rich in chlorogenic acid and rutin.

### 2.8. Comparative Metabolomic Profile of B. crataegina Extracts by LC–QTOF–MS

Untargeted LC–QTOF–MS profiling revealed 191 metabolites that were detected in at least two main extracts of *Berberis crataegina* DC. (Table S1). Flavonoids and flavonoid glycosides constituted the largest phytochemical class, accounting for 26.7% of these metabolites (51 metabolites), followed by fatty acids and lipid derivatives (15.7%, 30 metabolites), phenolic and organic acids (13.1%, 25 metabolites), and terpenoids (12.0%, 23 metabolites). Alkaloids and other phenolic compounds each accounted for 5.2% (10 metabolites each), while carbohydrates and glycosylated compounds represented 4.7% (9 metabolites). Amino acids and their derivatives and catechins/proanthocyanidins each comprised 4.2% (8 metabolites each), followed by lignans (3.7%, 7 metabolites), coumarins (2.6%, 5 metabolites), iridoids and iridoid glycosides (1.6%, 3 metabolites), and anthocyanins/betacyanins (1.0%, 2 metabolites).

In addition, metabolites detected exclusively in a single main extract of *B. crataegina* and not found in any of the other extracts are presented in Table 12. These extract-specific metabolites highlight the distinctive phytochemical profiles of the respective extracts and facilitate the comparison of chemical diversity among different plant parts.

**Figure 3:**
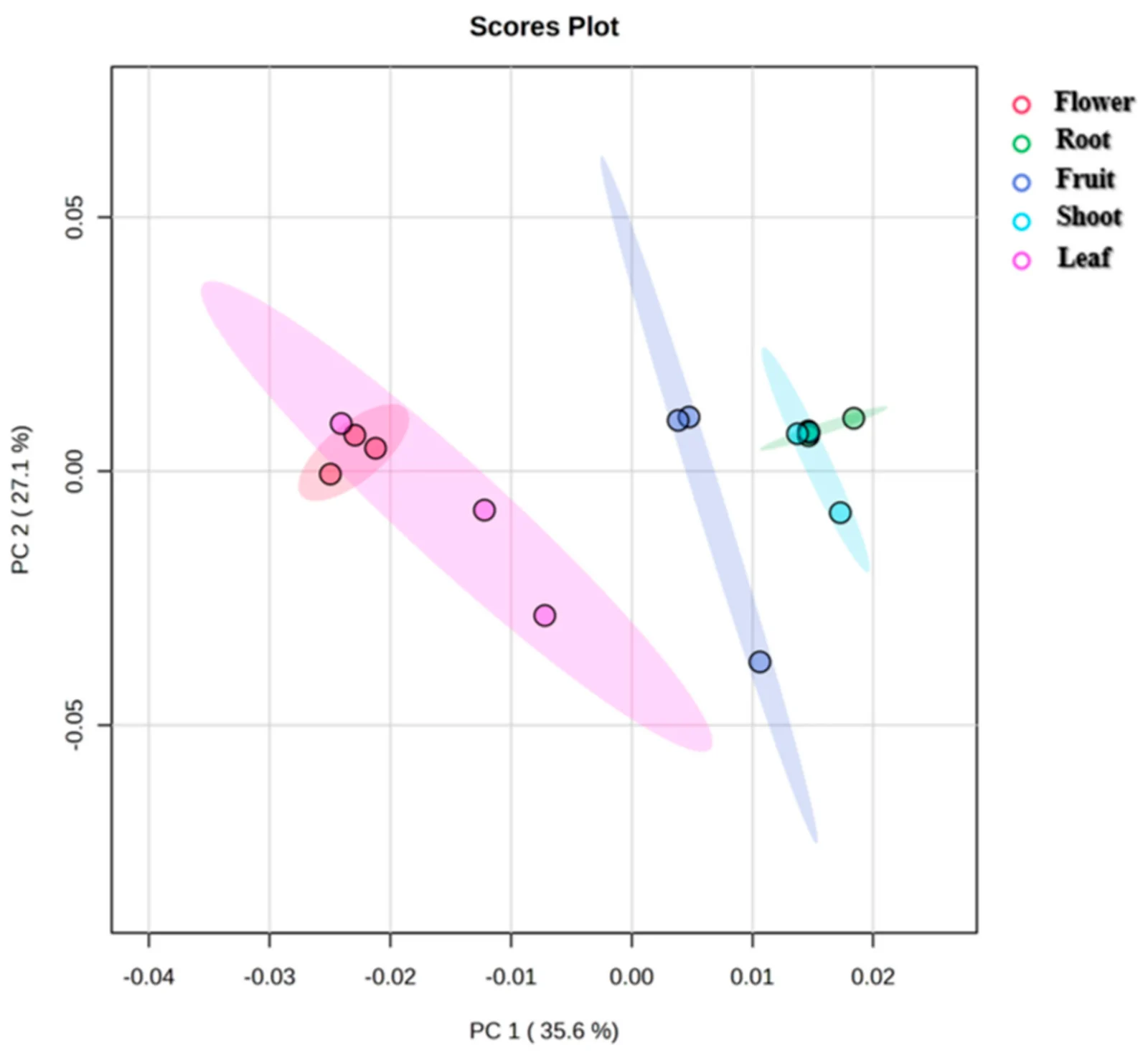
PCA score plot generated using the LC–QTOF–MS data of the main extracts of *Berberis crataegina* DC. The colored shaded areas represent the 95% confidence regions for the respective sample groups.

The principal component analysis (PCA) score plot (Figure 3) demonstrated the distribution of the main extracts of *B. crataegina* according to their metabolomic profiles. Notably, the fruit extract formed a distinct cluster, clearly separated from the other extracts, indicating its unique metabolomic profile.

The relationship between the phytochemical metabolites detected in the fruit extract and α-glucosidase inhibitory activity was evaluated using Pearson correlation analysis, and the results are presented in Table 13. Several phenolic acids, flavonoids, flavonoid glycosides, and tannin-related compounds exhibited statistically significant positive correlations with α-glucosidase inhibitory activity. In particular, quercetin, ellagic acid, trans-ferulic acid, rutin, and baicalein 7-O-glucuronide were distinguished by their high correlation coefficients. Moreover, several metabolites detected exclusively in the fruit extract, including 4″-O-acetylmyricitrin, apigenin 7-O-β-D-glucuronopyranoside methyl ester, β-glucogallin, chrysoeriol 4′,7-diglucuronide, isorhamnetin 3-(6″-malonylglucoside), kaempferol 3-(6-acetylgalactoside), kaempferol 3-glucuronide, luteolin 3′-methyl ether 7-glucuronosyl-(1→2)-glucuronide, and padmatin 3-glucoside, also showed strong positive correlations with the activity. These findings suggest that these phytochemical metabolites may contribute to the α-glucosidase inhibitory effect of the fruit extract.

The “+” symbol indicates metabolites detected exclusively in the fruit extract, whereas the “−” symbol indicates metabolites that were not fruit-specific and were also detected in at least one other extract. All listed correlations were positive and statistically significant at *p* < 0.05. The phytochemical metabolites detected by LC–QTOF–MS analysis of the fractions obtained from the fruit extract by RP-VLC, together with their distribution across the fractions, are presented in Table 14.

## 3. Discussion

The present study provides a comparative evaluation of the phytochemical composition and in vitro diabetes-related biological activities of the leaves, flowers, shoots, roots, and fruits of *B. crataegina*. Among the investigated plant parts, the fruit extract exhibited the most consistent biological profile, combining the highest total phenolic and flavonoid contents and the strongest DPPH radical-scavenging and α-glucosidase inhibitory activities with a cytoprotective effect in high-glucose-exposed SH-SY5Y cells. The separation of the fruit extract from the remaining extracts in the PCA score plot further indicated that this biological activity was accompanied by a distinct metabolomic composition.

Previous phytochemical studies on *B. crataegina* have characterized selected plant parts and compound classes. Quantitative HPLC analysis of the roots, bark, and stems identified berberine and berbamine as major alkaloids, whereas analysis of the fruits revealed several phenolic constituents, including chlorogenic, syringic, gallic, caffeic, sinapic, and vanillic acids, together with rutin and apigenin-7-glucoside. A broad phytochemical diversity, particularly comprising alkaloids, flavonoids, and phenolic compounds, has also been reported for other *Berberis* species growing in Türkiye, including *B. vulgaris* and *B. integerrima*. In *B. vulgaris*, various alkaloids have been reported from the roots, while its fruits have been shown to contain several phenolic constituents, including protocatechuic acid, 5-caffeoylquinic acid, caffeic acid, catechin, quercitrin, afzelin, and isorhamnetin-3-glucoside. Likewise, diverse alkaloids have been identified in the leaves, branches, and roots of *B. integerrima*. Beyond these phytochemical investigations, several studies have examined the roots and fruits of *B. vulgaris* and *B. integerrima* in relation to diabetes, whereas only limited evidence is available for *B. cretica* in this context. Although different plant parts of *B. crataegina* have been reported to be traditionally used against diabetes, a comprehensive comparative evaluation integrating the phytochemical profiles and diabetes-related biological activities of its different plant parts, particularly in the context of diabetic neuropathy and diabetes-related complications, had remained lacking prior to the present study [15,29,30,31,32,33].The fruit extract exhibited the highest total phenolic (267.11 mg GAE/g extract; Table 2) and total flavonoid (46.55 mg QE/g extract; Table 3) contents, together with the strongest DPPH radical-scavenging activity (RC_50_ = 60.11 µg/mL; Table 4). In the CUPRAC assay, the fruit extract also showed high antioxidant capacity (182.05 mg GAE/g extract; Table 5), although the highest value was observed for the shoot extract (243.34 mg GAE/g extract; Table 5). Previous studies have likewise identified *B. crataegina* fruits as rich sources of phenolic compounds with remarkable antioxidant activity. Charehsaz et al. reported that *B. crataegina* fruit extract exhibited strong antioxidant activity and protected DNA integrity, while Eroğlu et al. demonstrated high phenolic contents and antioxidant capacities in fruits collected from different geographical regions. Our findings are consistent with these previous reports [23,24].

A particularly important finding was the marked α-glucosidase inhibitory activity of the fruit extract, whereas the leaf, flower, root, and shoot extracts showed no or only limited activity. The fruit extract produced concentration-dependent α-glucosidase inhibition, increasing from 45.57 ± 0.39% at 100 µg/mL to 73.46 ± 0.77% at 1600 µg/mL, with an IC_50_ value of 140.45 µg/mL. Under the same experimental conditions, acarbose exhibited an IC_50_ value of 504.11 µg/mL (Table 6). Recently, Günbatan et al. evaluated the methanolic extract prepared from the aerial parts and fruits of *B. crataegina* and reported 53.50 ± 4.26% α-glucosidase inhibition at 300 µg/mL with an IC_50_ value of 2667.33 ± 433.37 µg/mL [34]. Although direct comparisons should be interpreted cautiously because of differences in the plant material, extraction procedures, and assay conditions, the stronger activity observed for the fruit extract in the present study suggests that the fruit may represent the principal contributor to the α-glucosidase inhibitory potential of *B. crataegina*.

The LC-QTOF-MS analysis revealed broad phytochemical diversity across the different parts of *B. crataegina*. Among the 191 metabolites detected in at least two extracts, flavonoids and flavonoid glycosides represented the largest class, followed by fatty acids and lipid derivatives, phenolic and organic acids, and terpenoids. Nevertheless, each plant part also showed a characteristic set of extract-specific metabolites (Table 12). The leaf extract was distinguished by the presence of the benzylisoquinoline alkaloid (S)-reticuline, several flavonoid glycosides, and secologanin, whereas the flower extract contained mainly kaempferol- and quercetin-derived glycosides together with a coumarin derivative. The shoot extract showed a more diverse profile comprising flavonoids, an anthocyanin, hydroxycinnamic acid derivatives, and coumarins, while only a limited number of extract-specific flavonoid and coumarin derivatives were detected in the root extract. These findings show that the phytochemical composition of *B. crataegina* varies considerably among plant organs, as previously reported for other *Berberis* species, in which alkaloids and phenolic compounds displayed organ-dependent distributions [35].

Among the investigated plant parts, the fruit exhibited the most distinctive metabolomic profile and formed a clearly separated cluster in the PCA score plot (Figure 3), indicating a metabolite composition substantially different from those of the vegetative and underground organs. Most metabolites detected exclusively in the fruit were flavonoids and their glycosylated, glucuronidated, acetylated, or malonylated derivatives, including apigenin 7-O-β-D-glucuronopyranoside methyl ester, chrysoeriol 4′,7-diglucuronide, isorhamnetin 3-(6″-malonylglucoside), and 4″-O-acetylmyricitrin, together with tannin-related compounds such as β-glucogallin and methylated ellagic acid derivatives (Table 12). This chemically distinctive profile agrees with previous reports describing *B. crataegina* fruits as rich sources of polyphenolic and anthocyanin-related constituents [24].

Targeted HPLC-DAD analysis confirmed the presence of chlorogenic acid, rutin, caffeic acid, protocatechuic acid, quercetin, and quercetin-3-O-glucoside in the fruit extract, with chlorogenic acid and rutin being the most abundant quantified compounds (Table 11). The validation results confirmed that the developed RP-HPLC-DAD method is sensitive, precise, and accurate for the simultaneous quantification of the target phenolic compounds in the plant extract. The low LOD and LOQ values obtained reflected the method’s capability to detect and quantify these compounds even at trace levels, while the observed RSD% and recovery values fell within acceptance criteria (RSD ≤ 15%, or ≤20% near the LOQ) that have been widely adopted for HPLC method validation, including in studies on phenolic compounds in plant matrices [36]. Minor variations in recovery and precision observed for certain compounds are attributable to the inherent complexity of the plant extract matrix and do not compromise the overall reliability of the method for phytochemical characterization. Several of these phenolic compounds, including chlorogenic acid, catechins, rutin, quercetin derivatives, and procyanidins, have previously been associated with α-glucosidase inhibitory activity, although their inhibitory potency depends on compound structure and assay conditions [37,38,39,40]. Thus, these metabolites likely contribute to the biological activity of the fruit extract. However, considering their quantified concentrations and the complexity of the untargeted metabolomic profile, the observed activity is unlikely to be attributable to a single compound.

Mechanistically, chlorogenic acid has been reported to inhibit α-glucosidase through a reversible mixed-type mechanism involving hydrogen bonding and hydrophobic interactions, accompanied by conformational changes in the enzyme [41]. Rutin and structurally related flavonoids can also interact directly with α-glucosidase and contribute to enzyme inhibition [42]. Quercetin-3-O-glucoside has also demonstrated direct α-glucosidase inhibitory activity, although glycosylation may influence enzyme binding and inhibitory potency relative to the corresponding aglycone [43]. Thus, the presence of chlorogenic acid, rutin, quercetin, and quercetin glycosides may partly explain the α-glucosidase inhibitory activity of the fruit extract.

The correlation analysis further supported this interpretation. Quercetin, ellagic acid, trans-ferulic acid, rutin, baicalein 7-O-glucuronide, and several fruit-specific flavonoid conjugates were positively associated with α-glucosidase inhibition (Table 13). These findings suggest that multiple phenolic constituents may contribute to the observed activity. However, these correlations indicate associations and do not establish causal relationships.

The fractionation results also supported a multicomponent contribution to α-glucosidase inhibition. Although several fractions retained appreciable activity, none reproduced the concentration-dependent inhibitory profile of the crude fruit extract (Table 7). This finding suggests that the activity is distributed among multiple constituents rather than arising from a single dominant inhibitor. Taken together, the phytochemical, correlation, and fractionation analyses indicate that the α-glucosidase inhibitory activity of the fruit extract most likely results from the combined contribution of chemically diverse phenolic constituents. Confirmation of the contribution of individual compounds and their interactions will require further bioactivity-guided isolation studies.

The cell-based findings further differentiated the biological profiles of the investigated plant parts. The fruit extract showed no marked cytotoxicity over the tested concentration range of 25–400 µg/mL, maintaining cell viability between 87.24% and 102.59%. The leaf and shoot extracts also showed relatively low cytotoxicity, whereas the root extract produced a marked concentration-dependent reduction in cell viability, decreasing viability from 71.40% at 25 µg/mL to 4.40% at 400 µg/mL (Table 8). Our LC-QTOF-MS profiling revealed several isoquinoline alkaloids in *B. crataegina*, consistent with previous phytochemical investigations reporting berberine and related protoberberine alkaloids in the roots and bark of this species [31,44]. The pronounced cytotoxicity of the root extract may therefore be partly associated with its alkaloid composition. This interpretation is also consistent with the concentration-dependent cellular effects reported for isoquinoline alkaloids; for example, berberine reduced SH-SY5Y cell viability at 30 µM, although lower concentrations showed little toxicity [45,46].

Regarding the fruit extract, berberine itself was not putatively annotated, whereas several related isoquinoline alkaloids were detected at MSI Level 2 (Supplementary Table S1). However, these alkaloids were not quantitatively determined; therefore, their concentrations and toxicological relevance cannot be established from the present analysis. Given the recognized safety concerns and potential herb–drug interactions associated with berberine and related isoquinoline alkaloids, targeted quantitative alkaloid analysis and further toxicological studies are required before the safety profile of the fruit extract can be established.

Among the extracts evaluated at non-cytotoxic concentrations, the fruit extract showed the clearest protective response under high-glucose conditions. Exposure to 50 mM glucose reduced SH-SY5Y cell viability, whereas the fruit extract at 25 µg/mL increased viability by approximately 22% relative to the injured control and restored it to a level close to the normal control (Table 9). In contrast, the other extracts did not produce a comparable improvement within the tested concentration ranges. The high phenolic content, antioxidant capacity, and flavonoid-rich metabolomic profile of the fruit extract are consistent with this cytoprotective response. Since phytochemicals have been reported to exert protective effects at low doses by activating adaptive stress responses and antioxidant defense mechanisms, whereas their efficacy may diminish or cellular stress may increase at higher doses, polyphenolic extracts may exhibit a nonlinear dose–response relationship with more pronounced protective effects within the low-to-moderate dose range; however, further studies including lower concentrations are required to confirm this relationship, identify the metabolites responsible for the observed effect, and clarify the underlying mechanisms [47,48].

The major phenolic constituents detected in the fruit extract may also contribute to its antioxidant and cytoprotective effects through mechanisms relevant to hyperglycemia-induced neuronal injury. In experimental diabetic neuropathy, chlorogenic acid reduced oxidative stress and inflammatory mediators and improved behavioral and histopathological outcomes [49]. Rutin alleviated diabetic neuropathy by activating Nrf2-mediated antioxidant defenses and reducing oxidative stress [50]. Quercetin preserved mitochondrial function in experimental diabetic peripheral neuropathy through activation of the AMPK/PGC-1α pathway [51]. In SH-SY5Y cells, quercetin also increased the expression of mitochondrial biogenesis-related proteins, including SIRT1, PGC-1α, and TFAM, while reducing ROS production and apoptosis [52]. These mechanisms provide biological plausibility for the antioxidant and cytoprotective activities of the fruit extract. However, the individual contributions and possible interactions of these constituents were not directly investigated in the present study.

Chronic hyperglycemia is a major driver of DN and induces oxidative and nitrosative stress through several interconnected metabolic pathways, including the polyol, AGE, PKC, hexosamine, and PARP pathways. Excessive generation of reactive oxygen and nitrogen species can impair mitochondrial function and activate stress-responsive signaling pathways, including MAPKs and NF-κB, thereby promoting inflammatory and cellular injury. In addition, AGE accumulation and subsequent RAGE activation further enhance oxidative stress and NF-κB-mediated signaling, while persistent mitochondrial dysfunction may promote the release of pro-apoptotic factors such as cytochrome c and subsequent caspase activation, ultimately contributing to neuronal apoptosis. These interconnected mechanisms are considered central to hyperglycemia-induced neuronal damage in DPN. Among the cellular models used to investigate glucose-related neuronal alterations, SH-SY5Y neuroblastoma cells are widely employed because they exhibit neuronal characteristics and, under high-glucose conditions, show alterations comparable to those reported in dorsal root ganglion neurons and Schwann cells, including changes in intracellular signaling, nitric oxide-related pathways, and Na^+^/K^+^-ATPase activity. Accordingly, the high-glucose-treated SH-SY5Y model used in the present study represents a simplified in vitro system reflecting the hyperglycemia-associated neuronal stress component of DN. The increased cell viability observed following *B. crataegina* fruit extract treatment therefore supports a cytoprotective effect under high-glucose conditions; however, its effects on oxidative stress, AGE/RAGE–NF-κB signaling, mitochondrial function, and apoptotic pathways were not directly investigated and require further mechanistic evaluation [5,10,53,54].

The findings of this study should be interpreted in light of several limitations. The high-glucose-exposed SH-SY5Y model represents only a simplified in vitro model of diabetes-related neuronal stress, and the observed metabolite–bioactivity correlations do not establish causality. In addition, metabolite identification in the untargeted LC–QTOF–MS analysis was based on putative annotation, and the mechanisms underlying the observed cytoprotective effects were not directly investigated. Finally, the biological findings require further validation in appropriate in vivo models.

Overall, the integration of phytochemical profiling with biological evaluation highlights the edible fruit of *B. crataegina* as the most promising source of antidiabetic bioactive constituents and provides a rational basis for its further pharmacological and functional food investigations.

## 4. Materials and Methods

### 4.1. Plant Material

*B. crataegina* fruits were collected from Kızılcahamam, Ankara, Türkiye, in October 2020, whereas vegetative parts (leaves, flowers, shoots, and roots) were collected from the same locality in May 2024. Voucher specimens corresponding to these collections were deposited in the Herbarium of Hacettepe University Faculty of Pharmacy (HUEF) under accession numbers HUEF 20137 and HUEF 24240, respectively.

### 4.2. Preparation of Crude Extracts

Fresh plant material was shade-dried and separated into individual parts (leaves, flowers, shoots, roots, and fruits). Each part was finely powdered using a mechanical grinder. The powdered samples were transferred into extraction flasks and extracted three times with 70% ethanol (2 h for each extraction) in a water bath at 40 °C. The combined extracts were filtered and concentrated under reduced pressure using a rotary evaporator, then subjected to lyophilization.

For all biological assays, test solutions were prepared by weighing the dried extracts, and the concentrations were expressed as dry extract weight per unit volume (µg/mL or mg/mL).

### 4.3. Total Phenolic Content Determination

The total phenolic content was assessed using a slightly modified Folin–Ciocalteu assay. Appropriately diluted extract samples or gallic acid standards were combined with Folin–Ciocalteu reagent previously diluted in distilled water. After the addition of 7.5% (*w*/*v*) Na_2_CO_3_ solution, the reaction mixtures were kept in the dark at room temperature for 2 h. Absorbance was subsequently recorded at 765 nm using a SPECTROstar Nano spectrophotometer (BMG LABTECH GmbH, Ortenberg, Germany). Measurements were carried out in triplicate, and the results were reported as milligrams of gallic acid equivalents per gram of extract (mg GAE/g extract) [55,56].

### 4.4. Total Flavonoid Content Determination

Total flavonoid content was determined using the aluminum chloride colorimetric method, which is based on the complex formation of flavonoid compounds with Al(III) ions. 10 μL of a 2% aluminum chloride solution (AlCl_3_; prepared in a methanol/acetic acid mixture, 95/5, *v*/*v*) was added to 100 μL of the sample. Then, 140 μL of the methanol/acetic acid mixture (95/5, *v*/*v*) was added. After the prepared mixture was left to stand at room temperature for 30 min, the absorbance value was measured at 415 nm. Absorbance measurements were corrected by subtracting the initial absorbance of the sample at 415 nm. All measurements were performed in triplicate. The calibration curve was created using quercetin standard solutions and the total flavonoid content results were expressed as mg quercetin equivalent per gram of extract [57,58].

### 4.5. Determination of Antioxidant Capacity

#### 4.5.1. DPPH Radical Scavenging Activity Assay

The DPPH radical scavenging capacities of the samples were determined spectrophotometrically based on the decrease in absorbance of a 2,2-diphenyl-1-picrylhydrazyl (DPPH) methanolic solution. DPPH is a stable free radical that is reduced to its non-radical form upon reaction with antioxidant compounds capable of donating hydrogen atoms [59].

Briefly, 200 μL of sample solutions prepared at different concentrations in methanol were mixed with DPPH methanolic solution. Following incubation for 30 min, the absorbance corresponding to the remaining DPPH radical content was measured at 520 nm using a microplate reader. The DPPH radical scavenging activity of the samples and the positive control, ascorbic acid, was evaluated by comparing the decrease in absorbance of the DPPH solution after sample treatment with that of a control solution containing only DPPH and solvent. The percentage of DPPH radical scavenging activity was calculated using the following equation [55,60]:

DPPH radical scavenging activity (%) = [(A_control_ − A_sample_)/A_control_] × 100

where A_control_ is the absorbance of the control solution and A_sample_ is the absorbance measured in the presence of the test sample.

#### 4.5.2. CUPRAC Assay

The assay was performed in 96-well microplates. Briefly, 50 μL of copper(II) chloride (CuCl_2_) solution (1.0 × 10^−2^ M), 50 μL of neocuproine solution (7.5 × 10^−3^ M), 50 μL of ammonium acetate (NH_4_Ac) buffer (1.0 M), and 50 μL of sample solutions prepared at different concentrations (31.25, 62.5, and 125 μg/mL) were added sequentially to each well. The final volume was adjusted to 250 μL with distilled water. Following incubation at room temperature for 30 min, absorbance was measured at 450 nm. The CUPRAC assay is based on the reduction of Cu^2+^ to Cu^+^ by antioxidant compounds, resulting in the formation of a colored Cu^+^–neocuproine complex with increased absorbance. All experiments were performed in triplicate. Gallic acid was used as the reference compound, and the results were expressed as mg gallic acid equivalents (GAE) per gram of extract using a calibration curve [61,62].

### 4.6. α-Glucosidase Inhibitory Activity Assay

The α-glucosidase inhibitory activity was determined using a modified version of previously described methods [63,64]. All experiments were carried out in 96-well microplates. Each well was loaded with 50 μL of potassium phosphate buffer (0.1 M, pH 6.9), 10 μL of α-glucosidase enzyme solution (1 U/mL; Sigma-Aldrich St. Louis, MO, USA,) prepared in the same buffer, and various concentrations of the test samples dissolved in a dimethyl sulfoxide (DMSO): phosphate buffer mixture (1:1, *v*/*v*). After a pre-incubation period of 5 min at 37 °C, the enzymatic reaction was initiated by adding 20 μL of 3 mM p-nitrophenyl-α-D-glucopyranoside (pNPG; Cayman, Ann Arbor, USA) prepared in phosphate buffer. The reaction mixture was then incubated at 37 °C for 30 min. To terminate the reaction, 50 μL of 0.1 M sodium carbonate solution prepared in phosphate buffer was added to each well. Absorbance was measured at 405 nm using a microplate spectrophotometer. Acarbose (Glucobay^®^, Bayer, Istanbul, Türkiye) was employed as the positive control, while the DMSO: phosphate buffer mixture (1:1, *v*/*v*) served as the negative control. The percentage inhibition of α-glucosidase was calculated using the following equation:

α-Glucosidase inhibition % = [(A_control_ − A_sample_)/A_control_] × 100

A_control_: The absorbance value obtained when the assay was performed with the negative control.

A_sample_: The absorbance value obtained after the assay was performed with the test sample (extracts or positive control).

### 4.7. Cell Culture Studies on Protective Effects in a High-Glucose-Induced Diabetic Neuropathy Model

#### 4.7.1. Determination of In Vitro Neurotoxicity and Safe Concentration Range in SH-SY5Y Neuroblastoma Cells

To determine the safe concentration range to be used in the subsequent DN model and to evaluate potential in vitro cytotoxicity, the cytotoxic effects of the *B. crataegina* crude extracts were assessed in SH-SY5Y neuroblastoma cells using the resazurin assay.

This method is based on the reduction of resazurin to highly fluorescent resorufin as a result of the metabolic activity of viable cells [65]. Since non-viable cells lose their metabolic capacity, they do not produce a fluorescence signal.

Adherent SH-SY5Y cells were seeded into 96-well plates at a total density of 5 × 10^5^ cells per plate and incubated overnight to allow cell attachment. Subsequently, the attached cells were treated with different concentrations of the extracts (25–400 µg/mL). Doxorubicin was used as a positive control at concentrations of 0.001, 0.01, 0.1, 1, and 10 µM.

After 72 h of incubation, 0.01% (*w*/*v*) resazurin solution (Sigma-Aldrich, St. Louis, MO, USA ) was added, and the cells were further incubated at 37 °C for 4 h. Fluorescence intensity was measured using a SpectraMax iD3 Multi-Mode Microplate Detection Platform (Molecular Devices, San Jose, CA, USA ) at excitation and emission wavelengths of 544 and 590 nm, respectively. Each experiment was performed independently at least three times.

Concentration–response curves were generated, and concentrations maintaining cell viability above 80% were considered safe for further experiments.

#### 4.7.2. Assessment of SH-SY5Y Cell Viability Under High-Glucose Conditions: In Vitro DN Model

SH-SY5Y neuroblastoma cells grown in DMEM containing 25 mM glucose were seeded into 96-well plates at a total density of 2 × 10^5^ cells per plate and incubated overnight to allow cell attachment. Subsequently, 50 mM glucose was added to the adherent cells to induce oxidative stress and establish the in vitro diabetic neuropathy model. The *B. crataegina* crude extracts were applied together with the high-glucose solution within the concentration ranges previously determined to be safe.

After 24 h of incubation, resazurin solution was added to the wells, and the cells were incubated at 37 °C. The resulting fluorescence intensity was measured using a microplate reader.

The protective effects of the tested samples against high-glucose-induced loss of cell viability were evaluated by comparison with the control group containing 25 mM glucose and the negative control group treated with 50 mM glucose. Cell viability values were calculated by considering the 25 mM glucose control group as 100%, and the protective effects of the samples were interpreted based on the increase in viability compared with the 50 mM glucose group [66].

### 4.8. Bioactivity-Guided Fractionation of Fruit Extract by Reversed-Phase Vacuum Liquid Chromatography

The fruit extract, which showed the highest α-glucosidase inhibitory activity among the crude extracts, was fractionated by RP-VLC using a bioactivity-guided fractionation approach.

Briefly, 40 g of LiChroprep C18 reversed-phase silica gel was suspended in approximately 200 mL of methanol and packed into the column. The column was conditioned sequentially with 75%, 50%, and 25% methanol, followed by 100% water. The fruit extract (4.4342 g) was dissolved in a suitable solvent system and applied onto the column by wet loading.

Elution was performed using a stepwise methanol gradient of 10%, 25%, 50%, and 75% MeOH. A total of 25 fractions were collected and analyzed by thin-layer chromatography (TLC). Fractions showing similar TLC profiles were combined and concentrated under reduced pressure, yielding seven subextracts, designated as Fr. A–Fr. G. These subextracts were further evaluated by TLC and tested for α-glucosidase inhibitory activity.

### 4.9. HPLC-DAD Analysis of the Fruit Extract

The fruit extract, which exhibited the highest α-glucosidase inhibitory activity among the crude extracts, was analyzed by HPLC-DAD to determine its qualitative and quantitative phenolic profile.

The fruit extract was prepared at a concentration of 10 mg/mL in 80% acetonitrile (ACN) and filtered through a 0.45 µm PTFE membrane filter prior to analysis. HPLC-DAD analyses were performed in triplicate using an Agilent 1260 LC system (Agilent Technologies, Santa Clara, CA, USA) equipped with a control unit, a G1311B quaternary LC pump, and a G7115A diode-array detector.

Chromatographic separation was carried out on an ACE 5 C18 column (150 mm × 4.6 mm, 5 µm, Advanced Chromatography Technologies Ltd., Aberdeen, UK) maintained at 25 °C, with an injection volume of 20 µL. The mobile phase consisted of solvent A, 80% ACN containing 0.1% formic acid, and solvent B, water containing 0.1% formic acid. Gradient elution was applied starting with 5% solvent A, which was gradually increased to 100% during the analysis. The total run time was 55 min, followed by a 5 min re-equilibration step to return the system to the initial conditions [67].

A targeted set of phenolic acids and flavonoids was investigated, including gallic acid, chlorogenic acid, vanillic acid, caffeic acid, p-coumaric acid, ferulic acid, ellagic acid, fumaric acid, rosmarinic acid, quinic acid, protocatechuic acid, protocatechuic aldehyde, p-hydroxybenzoic acid, catechin, epicatechin, epigallocatechin, rutin, quercetin-3-O-glucoside, luteolin-7-O-glucoside, apigenin-7-O-glucoside, hesperidin, myricetin, quercetin, luteolin, apigenin, kaempferol, and naringenin.

Quantification of the detected compounds was performed using calibration curves prepared at six different concentrations for each reference standard. Calibration curves were constructed based on the relationship between peak area and standard concentration.

#### Method Validation

The analytical method was validated for linearity, limit of detection (LOD), limit of quantification (LOQ), precision, and accuracy (recovery) in accordance with the ICH Q2(R1) guideline [68].

Linearity: Calibration curves for each phenolic compound were constructed using six concentration levels within the respective calibration ranges of the analytes (Table 11). Linearity was evaluated based on the coefficient of determination (R^2^) of the corresponding calibration equations.

LOD and LOQ: The LOD and LOQ were estimated using the standard deviation of the analytical response and the slope of the calibration curve according to the following equations: LOD = 3.3 × (SD/S) and LOQ = 10 × (SD/S) where SD is the standard deviation of the peak area obtained from six replicate injections of the lowest calibration standard, and S is the slope of the corresponding calibration curve.

Precision: The precision of the method was evaluated as intra-day and inter-day precision. The extract was analyzed in triplicate on the same day and in triplicate on a different day (n = 6), and the results were expressed as relative standard deviation (RSD%) of the determined analyte concentrations.

Accuracy (Recovery): The accuracy of the method was evaluated by a standard addition (spiking) procedure. The standard solutions were prepared at three concentration levels and the selected spike levels were designed to cover an appropriate concentration range while remaining within the established calibration ranges. Unspiked and spiked samples were analyzed under the same analytical conditions.

Recovery was calculated using the following equation:

Recovery (%) = [(Cspiked − Cunspiked)/Cadded] × 100

where Cspiked is the concentration determined in the spiked sample, Cunspiked is the concentration determined in the corresponding unspiked sample, and Cadded is the theoretical concentration of the analyte added by the standard addition.

### 4.10. LC–QTOF–MS-Based Untargeted Metabolomic Profiling

For LC–QTOF–MS analysis, 1 mg of each crude extract and fruit fraction was dissolved in 1 mL of methanol (9:1, *v*/*v*), and the samples were directly injected into the LC–QTOF–MS system without any further pretreatment.

Chromatographic separation was performed on a Zorbax C18 column (1 × 50 mm, 1.8 μm, 100 Å, Agilent Technologies, Santa Clara, CA, USA) using an Agilent 6530 LC–QTOF–MS system. Water containing 0.1% formic acid (A) and acetonitrile containing 0.1% formic acid (B) were used as the mobile phases, and separation was achieved under gradient elution conditions. The flow rate was set to 0.2 mL/min, and the injection volume was 2 μL.

Ionization was performed using an electrospray ionization (ESI) source in both positive and negative ionization modes. The capillary voltage and capillary temperature were set to 4000 V and 300 °C, respectively. MS/MS data were acquired in automatic MS/MS mode at a collision energy of 20 eV. Pooled QC samples were prepared and analyzed using the same methodology.

Raw data processing, including deconvolution, peak detection, and alignment, was performed using MS-DIAL 2.56 software. Feature detection cutoff was set to an amplitude of 500. For data collection, the MS1 and MS/MS tolerances were 0.01 and 0.025 Da. Alignment mass and time tolerance were 0.025 Da and 0.25 min. Mass silence width was 0.1 Da and sigma window value was 0.5. Metabolite annotation and structural prediction were carried out using MS-FINDER 3.04 software. The UNPD, KNAPSAcK, and PlantCyc natural product databases were used for metabolite identification. The mass tolerance was set to 10 ppm for both MS1 and MS/MS data, and only structures with a score above 6 were considered for further evaluation [69,70,71]. According to the Metabolomics Standards Initiative (MSI), the metabolites were classified as Level 2 putatively annotated compounds based on accurate-mass and MS/MS fragmentation data together with database-assisted and in silico structural matching. Principal component analysis was carried out using the Metaboanalyst 6.0 platform.

To explore the potential relationships between the metabolomic profiles and α-glucosidase inhibitory activity, Pearson correlation analysis was performed. Pearson’s correlation coefficient (r) was calculated between the relative abundance of each detected metabolite and α-glucosidase inhibitory activity. Statistical significance was set at *p* < 0.05.

### 4.11. Statistical Analysis

All experiments were performed using three independent biological replicates, with three technical replicates for each biological replicate, unless otherwise stated. The results are expressed as mean ± standard deviation (SD). Pearson’s correlation coefficient (r) was used to evaluate the relationships between phytochemical constituents and biological activities. Statistical significance was set at *p* < 0.05.

## 5. Conclusions

This study identifies the edible fruit of *B. crataegina* as the most promising plant part among those investigated for α-glucosidase inhibitory and cytoprotective activities. The integration of metabolomic profiling, HPLC-DAD analysis, correlation analysis, and fractionation suggested that these biological effects arise from the combined contribution of multiple phenolic constituents, including chlorogenic acid, rutin, quercetin, and related flavonoids, rather than a single dominant compound. However, synergistic interactions among these constituents were not experimentally demonstrated.

Overall, the findings indicate that the edible fruit of *B. crataegina* represents a valuable natural source of bioactive constituents relevant to diabetes and diabetes-related complications. Future studies should establish the underlying mechanisms of action, define appropriate markers for extract standardization and validate its biological activity through in vivo studies.

## Figures and Tables

**Table 1:** Data on the preparation and yields of the main extracts of *Berberis crataegina* DC.

Plant Part	Weight of Powdered Plant Material Used for Extraction (g)	Weight of Concentrated Extract Obtained (g)	Extraction Yield (% *w*/*w*)
Leaf	25.87	11.72	45.30
Flower	32.60	14.78	45.34
Shoot	39.71	4.33	10.90
Root	33.09	3.12	9.41
Fruit	200.0 (fresh)	49.25 (lyophilized)	24.62

**Table 2:** Total phenolic contents of the main extracts of *Berberis crataegina* DC. expressed as milligrams of gallic acid equivalents per gram of extract (mg GAE/g extract).

Plant Part	Total Phenolic Content (mg GAE/g Extract)
Leaf	153.93 ± 1.11
Flower	160.69 ± 3.14
Shoot	189.85 ± 2.57
Root	135.78 ± 5.24
Fruit	267.11 ± 2.10

**Table 3:** Total flavonoid contents of the main extracts of *Berberis crataegina* DC. expressed as milligrams of quercetin equivalents per gram of extract (mg QE/g extract).

Plant Part	Total Flavonoid Content (mg QE/g Extract)
Leaf	44.29 ± 6.46
Flower	15.42 ± 0.06
Shoot	21.35 ± 1.41
Root	45.33 ± 4.74
Fruit	46.55 ± 1.73

**Table 4:** RC_50_ values of the main extracts of *Berberis crataegina* DC. and ascorbic acid in the DPPH radical-scavenging assay.

Sample	RC_50_ (µg/mL)
Leaf extract	99.49
Flower extract	106.21
Shoot extract	98.77
Root extract	172.80
Fruit extract	60.11
Ascorbic acid	9.51

**Table 5:** CUPRAC antioxidant capacities of the main extracts of *Berberis crataegina* DC. expressed as milligrams of gallic acid equivalents per gram of extract (mg GAE/g extract).

Plant Part	CUPRAC Antioxidant Capacity (mg GAE/g Extract)
Leaf	219.06 ± 1.69
Flower	193.59 ± 6.19
Shoot	243.34 ± 1.13
Root	123.14 ± 1.13
Fruit	182.05 ± 5.63

**Table 6:** α-glucosidase inhibitory activities of the shoot and fruit extracts of *Berberis crataegina* DC. and acarbose.

Final Concentration (µg/mL)	Shoot Extract Inhibition (%)	Fruit Extract Inhibition (%)	Acarbose Inhibition (%)
100	ND	45.57 ± 0.39	26.92 ± 3.03
200	3.15 ± 0.92	56.52 ± 2.93	35.85 ± 4.77
400	6.64 ± 0.42	66.11 ± 3.80	47.16 ± 1.95
800	15.75 ± 0.81	72.04 ± 3.78	58.08 ± 2.45
1600	12.64 ± 0.15	73.46 ± 0.77	70.52 ± 3.84

Values are expressed as mean ± SD. ND: not detected.

**Table 7:** α-glucosidase inhibitory activities of fractions obtained from the fruit extract of *Berberis crataegina* DC.

Final Concentration (µg/mL)	Fr. A	Fr. B	Fr. C	Fr. D	Fr. E	Fr. F	Fr. G
100	50.81 ± 1.81	ND	3.40 ± 0.66	ND	23.21 ± 2.16	48.07 ± 1.06	51.02 ± 0.74
200	68.25 ± 0.23	ND	9.26 ± 0.66	ND	33.08 ± 0.87	42.80 ± 4.10	53.80 ± 1.04
400	69.00 ± 0.04	7.55 ± 3.17	16.91 ± 0.89	ND	43.33 ± 1.25	42.41 ± 0.49	54.15 ± 1.64
800	62.00 ± 1.64	20.02 ± 1.88	29.92 ± 0.31	ND	43.22 ± 0.04	24.23 ± 1.48	40.26 ± 1.72
1600	30.37 ± 4.15	38.35 ± 1.57	42.90 ± 2.15	ND	16.88 ± 1.33	ND	ND

Values are expressed as mean ± SD. Fr.: Fraction; ND: not detected.

**Table 8:** Cell viability (%) of SH-SY5Y cells treated with the main extracts of *Berberis crataegina* DC.

Extract	25 µg/mL	50 µg/mL	100 µg/mL	200 µg/mL	400 µg/mL
Leaf extract	79.23	88.50	81.38	83.09	80.02
Flower extract	95.39	83.11	83.73	82.18	80.15
Shoot extract	95.93	88.10	90.41	80.51	79.96
Root extract	71.40	58.88	44.43	20.62	4.40
Fruit extract	102.59	96.17	88.89	88.67	87.24

**Table 9:** Effects of the crude extracts of *Berberis crataegina* DC. on cell viability in the high-glucose-induced SH-SY5Y diabetic neuropathy model.

Extract	1.5625 µg/mL	3.125 µg/mL	6.25 µg/mL	12.5 µg/mL	25 µg/mL	50 µg/mL	100 µg/mL	200 µg/mL	400 µg/mL
Leaf	—	—	—	—	87.62	75.87	74.87	72.08	50.30
Flower	—	—	—	—	54.88	55.04	89.28	81.56	78.49
Shoot	—	—	—	—	89.75	82.93	80.13	89.60	82.63
Root	89.22	85.38	71.74	68.45	61.06	—	—	—	—
Fruit	—	—	—	—	122.83	91.39	79.24	73.42	62.10

Note: Cell viability values were calculated by setting the injured control group at 100%.

**Table 10:** Calibration parameters and validation data (LOD, LOQ, precision, and recovery) for the quantified phenolic compounds.

Phytochemical Compounds	Range (ppm)	LOD (ppm)	LOQ (ppm)	Intra-Day RSD% (Day1/Day2)	Intermediate Precision RSD%	Recovery (%)
Protocatechuic acid	1–100	0.0002	0.0006	0.45/1.37	7.67	82.01–95.93
Chlorogenic acid	1–100	0.0086	0.0260	1.66/1.13	4.10	90.75–101.47
Caffeic acid	1–100	0.0066	0.0201	0.15/0.19	1.91	90.95–104.37
Rutin	1–100	0.0167	0.0507	0.57/0.37	2.96	72.98–114.28
Quercetin-3-O-glucoside	0.25–25	0.0120	0.0364	0.80/1.59	4.43	103.27–124.72
Quercetin	1–100	0.0051	0.0155	1.69/1.11	2.37	98.80–104.90

**Table 11:** HPLC-DAD quantification of phenolic and flavonoid compounds detected in the fruit extract.

Phytochemical Compounds	Concentration Range (ppm)	Retention Time (min)	Quantification Wavelength (nm)	Calibration Curve Equation	R^2^	Content in Extract (mg/g) (Mean ± SD)
Protocatechuic acid	1–100	9.613	260	y = 112.7311x − 16.8136	0.9999	0.5395 ± 0.0124
Chlorogenic acid	1–100	15.408	320	y = 154.5722x − 66.7416	0.9999	5.8003 ± 0.1484
Caffeic acid	1–100	18.274	320	y = 149.5791x − 23.2838	0.9999	1.2039 ± 0.0026
Rutin	1–100	31.681	260	y = 43.2317x − 17.4150	0.9998	5.0736 ± 0.0576
Quercetin-3-O-glucoside	0.25–25	32.532	260	y = 77.6384x − 9.5982	0.9998	0.3082 ± 0.0188
Quercetin	1–100	41.063	260	y = 67.1133x + 2.3928	0.9999	0.4554 ± 0.0051

**Table 12:** Extract-specific phytochemical metabolites identified in the main extracts of *Berberis crataegina* DC. by LC–QTOF–MS analysis.

Plant Part	Main Class	Subclass	Extract-Specific Metabolite
Leaf	Alkaloids	Benzylisoquinolines	(S)-Reticuline
Leaf	Other	Long-chain fatty acids	Hexadeca-4,7,10,13-tetraenoic acid
Leaf	Flavonoids	Flavonoid O-glycosides	Isovitexin 4′-O-glucoside 2″-O-arabinoside/Vaxarin
Leaf	Flavonoids	Flavonoid 7-O-glycosides	6-Methoxykaempferol 3,7-bis(3-acetylrhamnoside)
Leaf	Flavonoids	Flavonoid 3-O-glycosides	Rhamnocitrin 3-rutinoside
Leaf	Terpenoids	Terpene glycosides	Secologanin
Flower	Flavonoids	Flavonoid 3-O-p-coumaroyl glycosides	3″,6″-Di-O-p-coumaroyltrifolin
Flower	Flavonoids	Flavonoid 3-O-glycosides	Kaempferide 3-galactoside
Flower	Flavonoids	Flavonoid 7-O-glycosides	Kaempferol 3-sophoroside 7-glucoside
Flower	Flavonoids	Flavonoid 3-O-glycosides	Quercetin 3-glucosyl-(1→6)-galactoside
Flower	Flavonoids	Flavonoid 3-O-glucuronides	Quercetin 3-O-glucuronide
Flower	Coumarins	Coumarins and derivatives	Ferulagol B
Shoot	Anthocyanins	Anthocyanidin 3-O-glycosides	Cyanidin 3-O-rutinoside
Shoot	Phenolic acids	Hydroxycinnamic acids	3-(3,4-Dihydroxy-5-methoxyphenyl)-2-propenoic acid
Shoot	Flavonoids	Flavonoid 7-O-glycosides	Astragalin 7-rhamnoside
Shoot	Flavonoids	Flavonoid 7-O-glycosides	Hesperidin
Shoot	Flavonoids	3-O-Methylated flavonoids	Isokaempferide/kaempferol 3-methyl ether
Shoot	Flavonoids	Flavones	Luteolin
Shoot	Flavonoids	Flavonoid 7-O-glycosides	Robinin
Shoot	Coumarins	Angular pyranocoumarins	Etuliacoumarin
Shoot	Coumarins	Coumarins and derivatives	Herniarin
Root	Flavonoids	Flavonoid 7-O-glycosides	Acacetin 7-glucoside/apigenin 4′-methyl ether 7-glucoside
Root	Coumarins	Coumarins and derivatives	Coumarin
Fruit	Other	Long-chain fatty acids	Heptadecanoic acid
Fruit	Phenolic acids	Phenylpropanoic acids	3-(3,4-Dihydroxyphenyl)lactic acid
Fruit	Flavonoids	Flavonoid 3-O-glycosides	(2R,3R)-Taxifolin 3-xyloside
Fruit	Flavonoids	Flavonoid 3-O-glycosides	4″-O-Acetylmyricitrin
Fruit	Flavonoids	3′-O-Methylated flavonoids	Chrysoeriol
Fruit	Flavonoids	Flavonoid 7-O-glucuronides	Chrysoeriol 4′,7-diglucuronide
Fruit	Flavonoids	Flavonoid 7-O-glucuronides	Chrysoeriol 7-O-glucuronide/luteolin 3′-methyl ether 7-glucuronide
Fruit	Flavonoids	Flavonoid 7-O-glycosides	Apigenin 7-O-(6″-O-acetylglucoside)
Fruit	Flavonoids	Flavonoid 7-O-glucuronides	Apigenin 7-O-β-D-glucuronopyranoside methyl ester
Fruit	Flavonoids	Flavonoid 7-O-glycosides	Hispidulin 7-glucoside
Fruit	Flavonoids	Flavonoid 3-O-glycosides	Isorhamnetin 3-(6″-malonylglucoside)
Fruit	Flavonoids	Flavonoid 3-O-glycosides	Kaempferol 3-(6-acetylgalactoside)
Fruit	Flavonoids	Flavonoid 3-O-glucuronides	Kaempferol 3-glucuronide
Fruit	Flavonoids	Flavonoid 7-O-glucuronides	Luteolin 3′-methyl ether 7-glucuronosyl-(1→2)-glucuronide
Fruit	Flavonoids	Flavonoid 3-O-glycosides	Padmatin 3-glucoside
Fruit	Tannins	Tannins	β-Glucogallin
Fruit	Tannins	Hydrolyzable tannins	3,3′,4-Tri-O-methylellagic acid 4′-O-β-D-glucopyranoside
Fruit	Tannins	Hydrolyzable tannins	3,3′-Di-O-methylellagic acid/3,3′-dimethoxyellagic acid

**Table 13:** Pearson correlation analysis between phytochemical metabolites detected in the fruit extract of *Berberis crataegina* DC. and α-glucosidase inhibitory activity.

Phytochemical Metabolite	Pearson’s *r*	*p* Value	Specific to the Fruit Extract
1-O-Caffeoylglucose	0.9216	0.0260	−
4″-O-Acetylmyricitrin	0.9572	0.0106	+
Apigenin 7-O-β-D-glucuronopyranoside methyl ester	0.9631	0.0085	+
Apigenin 7-O-glucoside	0.9392	0.0178	−
Baicalein 7-O-glucuronide	0.9062	0.0340	−
β-Glucogallin	0.9631	0.0085	+
Chrysoeriol 4′,7-diglucuronide	0.9631	0.0085	+
Ellagic acid	0.9586	0.0101	−
Isorhamnetin 3-(6″-malonylglucoside)	0.9508	0.0130	+
Kaempferol 3-(6-acetylgalactoside)	0.9569	0.0107	+
Kaempferol 3-glucuronide	0.9629	0.0085	+
Luteolin 3′-methyl ether 7-glucuronosyl-(1→2)-glucuronide	0.9622	0.0088	+
Padmatin 3-glucoside	0.9613	0.0091	+
Quercetin	0.9536	0.0119	−
Quercetin 3-O-glucoside	0.9211	0.0263	−
Rutin	0.9465	0.0148	−
trans-Ferulic acid	0.9646	0.0080	−

**Table 14:** Distribution of phytochemical metabolites identified by LC–QTOF–MS in RP-VLC fractions obtained from the fruit extract of *Berberis crataegina* DC.

Phytochemical Metabolite	Fr. A	Fr. B	Fr. C	Fr. D	Fr. E	Fr. F	Fr. G
Cinnamaldehyde	X	X	X	X	X	X	X
Cinnamic acid	X	X	X	X	X	X	X
Hyperoside	—	—	—	X	X	X	X
Caffeic acid	—	—	X	X	X	X	X
Chlorogenic acid	—	X	X	X	X	X	X
4-Hydroxycinnamic acid	—	X	X	X	X	X	X
Delphinidin glycoside	—	—	—	—	X	X	X
Syringetin 3-O-glucoside	—	—	—	—	X	X	X
Methyl syringate	—	—	—	—	X	X	X
Isoquercitrin	—	—	—	—	X	X	X
Naringenin 7-O-glucoside	—	—	—	—	X	X	X
Isoorientin 4′-O-glucoside 2″-O-*p*-hydroxybenzoate	—	—	—	—	X	X	X
(−)-Epicatechin	—	—	—	—	X	X	X
Luteolin	—	—	—	—	—	X	X
Apigenin 7-O-glucoside	—	—	—	—	—	X	X
Myricetin	—	—	—	—	—	X	X
Quercitrin	—	—	—	—	—	X	X
Kaempferide 3-galactoside	—	—	—	—	—	X	X
3′,4′,5,7-Tetrahydroxyflavanone	—	—	—	—	—	X	X
Quercetin	—	—	—	—	X	X	X
Isorhamnetin 3-O-rutinoside	—	—	—	—	X	X	X
Cyanidin 3-glucoside	—	—	—	—	X	X	X
Eriodictyol	—	—	—	—	X	X	X
4-Coumaryl alcohol	—	—	—	X	X	—	X
Apigenin	—	—	—	—	—	—	X

X indicates that the metabolite was detected in the corresponding fraction; — indicates that it was not detected.

## Data Availability

The data presented in this study are available from the corresponding author upon request.

## Supplementary Materials

The following supporting information can be downloaded at: https://www.mdpi.com/article/10.3390/ph19091365/s1; Table S1: Distribution of phytochemical metabolites among *B. crataegina* extracts by LC-QTOF-MS analysis.

## Abbreviations

The following abbreviations are used in this manuscript:

CUPRAC	Cupric reducing antioxidant capacity
DMSO	Dimethyl sulfoxide
DN	Diabetic neuropathy
DPPH	2,2-diphenyl-1-picrylhydrazyl
Fr.	Fraction
GAE	Gallic acid equivalents
GC-MS	Gas chromatography-mass spectrometry
HPLC-DAD	High-performance liquid chromatography–diode array detection
HUEF	Hacettepe University Faculty of Pharmacy
IC_50_	Half-maximal inhibitory concentration
LC-MS	Liquid chromatography-mass spectrometry
LC-QTOF-MS	Liquid chromatography–quadrupole time-of-flight mass spectrometry
ND	Not detected
NMR	Nuclear magnetic resonance
PCA	Principal component analysis
pNPG	p-nitrophenyl-α-D-glucopyranoside
QE	Quercetin equivalents
RC_50_	50% radical-scavenging concentration
RP-VLC	Reversed-phase vacuum liquid chromatography
TLC	Thin-layer chromatography
UV	Ultraviolet
