# Supplementary material for: Untargeted Metabolomic and Phytochemical Profiling of Berberis crataegina DC. Plant Parts: Antidiabetic Potential and Cytoprotection in High-Glucose-Exposed SH-SY5Y Cells
Source: Pharmaceuticals (Basel). 2026 Aug 28;19(9):1365. doi: 10.3390/ph19091365 (PMC13610044; doi:10.3390/ph19091365)
Supplement: Supplementary file 1 [file pharmaceuticals-19-01365-s001.zip › pharmaceuticals-4474948-supplementary.pdf]

Supplementary Table S1. Distribution of phytochemical metabolites among *B. crataegina* extracts by LC-QTOF-MS analysis

Phytochemical metabolite	Subclass	Leaf	Flower	Shoot	Root	Fruit
**ALKALOIDS**						
(+)-Berbamunin; Berbamunin	Benzylisoquinolines	✓	✓	✓	✓	✓
(-)-Isocorypalmine; Isocorypalmine; O10-Methylstepholidine; Tetrahydrocolumbamine	Protoberberine alkaloids and derivatives	✓	✓	✓	✓	✓
Canadine; Tetrahydroberberine; Tetrahydroumbellatine; Xanthopuccine	Protoberberine alkaloids and derivatives	–	✓	✓	✓	✓
N-(N-Methylcarbamoyl)-O-methylbulbocapnine	Aporphines	✓	✓	✓	✓	✓
Norcularine; (+)-Norcularine	Cularine alkaloids and derivatives	✓	✓	–	–	✓
Nortalifolin	Isoquinolones and derivatives	✓	✓	–	–	✓
Oxoanolobin	Aporphines	✓	–	–	✓	✓
Oxocularisine	Dibenzoxepins	✓	✓	✓	✓	✓
Oxonantenine	Aporphines	✓	✓	✓	✓	✓
Talmelatin; (+)-Talmelatin	Aporphines	–	–	✓	✓	–
**AMINO ACIDS AND DERIVATIVES**						
9,10-Dihydroexserohilone	Alpha-amino acids and derivatives	–	✓	✓	–	✓
Canavanine	L-alpha-amino acids	✓	✓	✓	✓	✓
L-5-Oxoproline	Alpha-amino acids and derivatives	✓	✓	✓	✓	✓
L-Glutamine	Alpha-amino acids	✓	✓	✓	✓	✓
Lenticin	L-alpha-amino acids	–	–	✓	✓	–
O-Carbamoyl-D-serine; O-Carbamoyl-D-serine	L-alpha-amino acids	✓	✓	✓	✓	✓
Pyroglutamic acid	Alpha-amino acids and derivatives	✓	✓	✓	✓	✓
S-Methyl-L-cysteine	Cysteine and derivatives	✓	✓	✓	✓	✓
**ANTHOCYANINS / BETACYANINS**						
Delphinidin-3-O-(6''-O-alpha-rhamnopyranosyl-beta-glucopyranoside)	Anthocyanidin-3-O-glycosides	✓	✓	✓	✓	✓
Amarantin	Betacyanins and derivatives	–	✓	–	–	✓
**PHENOLIC / ORGANIC ACIDS**						
(+)-Taraxafolin B	Coumaric acids and derivatives	✓	✓	✓	✓	✓
1-O-Feruloylglucose	Hydroxycinnamic acid glycosides	✓	✓	✓	✓	✓
1-O-Caffeoylglucose	Hydroxycinnamic acid glycosides	✓	✓	✓	✓	✓
1-O-p-Coumaroyl-beta-D-glucose	Hydroxycinnamic acid glycosides	✓	✓	–	–	–
1-O-Sinapoylglucose	Hydroxycinnamic acid glycosides	–	–	✓	✓	–
1-O-trans-p-Coumaroylglycerol	Coumaric acid esters	✓	✓	✓	✓	✓
2,5-dihydroxybenzoic acid	Hydroxybenzoic acid derivatives	✓	✓	✓	✓	✓
3,4-dihydroxybenzoic acid	Hydroxybenzoic acid derivatives	✓	–	✓	✓	✓
3-(4-hydroxyphenyl)prop-2-enoic acid	Hydroxycinnamic acids	–	✓	✓	–	✓
3Z-Hexenyl ferulate	Coumaric acids and derivatives	✓	✓	✓	✓	✓
[8]-Dehydrogingerdion	Hydroxycinnamic acids and derivatives	✓	✓	✓	✓	✓
Benzoic acid	Benzoic acids	✓	✓	✓	✓	✓
Chlorogenic acid	Quinic acids and derivatives	✓	–	✓	–	✓
D-(+)-Malic acid	Beta-hydroxy acids and derivatives	✓	✓	✓	✓	✓
Gentisic acid	Hydroxybenzoic acid derivatives	✓	✓	✓	✓	✓
Caffeic acid	Hydroxycinnamic acids	✓	✓	✓	✓	✓
L-Phenylalanine	Phenylalanine and derivatives	✓	✓	–	–	✓
L-Tryptophan	Indolyl carboxylic acids and derivatives	–	–	✓	✓	–

Phytochemical metabolite	Subclass	Leaf	Flower	Shoot	Root	Fruit
m-Hydroxycinnamic acid	Hydroxycinnamic acids	✓	✓	–	–	✓
Pectinolide A	Tricarboxylic acids and derivatives	–	✓	✓	–	✓
Quinic acid	Quinic acids and derivatives	✓	✓	✓	✓	✓
Sinapic acid	Hydroxycinnamic acids	–	–	✓	✓	–
Syringic acid	Gallic acid and derivatives	✓	✓	✓	✓	✓
trans-Ferulic acid	Hydroxycinnamic acids	✓	–	✓	✓	✓
Vanillic acid	M-methoxybenzoic acids and derivatives	✓	✓	✓	✓	✓
**PHENOLIC COMPOUNDS**						
1,2-Benzenediol	Catechols	–	✓	✓	✓	✓
1,3,5-Trihydroxybenzene	Phloroglucinols and derivatives	✓	✓	✓	✓	✓
3-Methoxybenzaldehyde	Benzoyl derivatives	✓	✓	✓	✓	✓
4-ethenylbenzen-1,3-diol	Resorcinols	✓	✓	✓	✓	✓
Caffeic acid 3-glucoside	Phenolic glycosides	✓	✓	✓	✓	✓
Embelin	P-benzoquinones	✓	✓	✓	✓	✓
Geranylhydroquinone	Prenylated hydroquinones	✓	✓	✓	✓	✓
Mascaroside	Naphthofurans	✓	✓	✓	✓	✓
Phenylacetaldehyde	Phenylacetaldehydes	✓	✓	✓	–	✓
Pyrocatechol	Catechols	✓	–	✓	–	✓
**FLAVONOIDS / FLAVONOID GLYCOSIDES**						
1-(2-hydroxy-4,6-dimethoxyphenyl)-3-(4-methoxyphenyl)propan-1-one	2'-hydroxy-dihydrochalcones	✓	✓	✓	✓	✓
3' 4' 5 7-tetrahydroxyflavanon	Flavanones	–	✓	✓	–	✓
5,7-Dihydroxy-6-C-methylflavanone 7-xylosyl-(1->3)-xyloside	Flavonoid 7-O-glycosides	✓	✓	✓	✓	✓
5,7-Dihydroxyflavone	Flavones	✓	–	✓	✓	✓
5-hydroxy-2-(4-hydroxyphenyl)-7-{[3,4,5-trihydroxy-6-(hydroxymethyl)oxan-2-yl]oxy}-3,4-dihydro-2H-1-benzopyran-4-one	Flavonoid 7-O-glycosides	✓	–	–	–	✓
Acuminatoside	Flavonoid 7-O-glycosides	–	–	✓	✓	–
Apigenin 7-O-beta-D-glucopyranosyl-(1->6)-beta-D-glucopyranoside	Flavonoid 7-O-glycosides	–	✓	✓	–	✓
Apigenin-7-O-glucoside	Flavonoid 7-O-glycosides	✓	–	✓	–	✓
Casticin 3'-glucoside	Flavonoid O-glycosides	–	✓	✓	✓	✓
Cosmosiin	Flavonoid 7-O-glycosides	–	✓	✓	–	✓
Diosmetin 7-O-beta-D-glucopyranoside	Flavonoid 7-O-glycosides	–	✓	✓	–	✓
Epicatechin 5-O-beta-D-glucopyranoside-3-benzoate; (2R,3R)-3-(Benzoyloxy)-2-(3,4-dihydroxyphenyl)-3,4-dihydro-7-hydroxy-2H-1-benzopyran-5-yl beta-D-glucopyranoside	Flavonoid O-glycosides	–	–	✓	✓	✓
Luteolin-4'-O-glucoside	Flavonoid O-glycosides	✓	✓	✓	–	✓
Luteolin-6-C-glucoside	Flavonoid C-glycosides	–	✓	–	–	✓
Luteolin-7-O-glucoside	Flavonoid 7-O-glycosides	–	✓	✓	–	✓
Pectolinarigenin 7-glucoside	Flavonoid 7-O-glycosides	–	–	✓	–	✓
Rutin	Flavonoid 3-O-glycosides	–	✓	✓	–	✓
Tricetin	Flavones	–	✓	✓	–	✓
Vitexin-2''-O-rhamnoside	Flavonoid 8-C-glycosides	–	–	✓	–	✓
2-(3,4-dihydroxyphenyl)-3,5,6,7-tetrahydroxy-4H-chromen-4-one	Flavonols	–	✓	–	–	✓
3,5,7-trihydroxy-4'-methoxyflavone	Flavonols	–	–	✓	–	✓
5,7-dihydroxy-2-(4-hydroxy-3-methoxyphenyl)-3-{[3,4,5-trihydroxy-6-(hydroxymethyl)oxan-2-yl]oxy}-4H-chromen-4-one	Flavonoid 3-O-glycosides	–	✓	✓	–	✓
[5',5']-Bisdihydroquercetin	Flavanonols	✓	–	–	–	✓

Phytochemical metabolite	Subclass	Leaf	Flower	Shoot	Root	Fruit
Epimedoside	Flavonoid 3-O-glycosides	–	–	✓	✓	–
Eupatolitin 3-galactoside	Flavonoid 3-O-glycosides	–	✓	✓	–	✓
Gossipin	Flavonoid 8-O-glycosides	–	✓	✓	–	✓
Isorhamnetin-3-O-rutinoside	Flavonoid 3-O-glycosides	–	✓	–	–	✓
Kaempferol	Flavonols	✓	✓	✓	–	✓
Kaempferol-3-O-rutinoside	Flavonoid 3-O-glycosides	✓	✓	–	–	✓
Quercetin	Flavonols	✓	✓	✓	–	✓
Quercetin 3-galactoside	Flavonoid 3-O-glycosides	✓	✓	✓	–	✓
Quercetin 4'-glucoside	Flavonoid O-glycosides	✓	✓	✓	–	✓
Quercetin-7-O-rhamnoside	Flavonoid 7-O-glycosides	✓	✓	✓	–	✓
Myricetin	Flavonols	–	✓	–	–	✓
Myricetin 3-galactoside	Flavonoid 3-O-glycosides	–	✓	✓	–	✓
Patuletin 7-galactoside	Flavonoid 7-O-glycosides	–	✓	✓	–	✓
Quercetagetin 7-methyl ether 3-(2'''-caffeoylglucosyl)-(1->2)-glucuronide	Flavonoid 3-O-glucuronides	–	✓	–	–	✓
Quercitrin	Flavonoid 3-O-glycosides	✓	✓	✓	–	✓
Rhamnazin 3-rutinoside	Flavonoid 3-O-glycosides	–	✓	✓	–	✓
Spinacetin 7-glucoside	Flavonoid 7-O-glycosides	✓	✓	–	–	✓
Tamarixetin 3-hexoside	Flavonoid 3-O-glycosides	✓	–	–	–	✓
Trifolin	Flavonoid 3-O-glycosides	✓	✓	✓	–	✓
Typhaneoside	Flavonoid 3-O-glycosides	✓	✓	✓	–	–
5,7,4'-Trihydroxy-6-methoxyisoflavone 4'-O-(6''-glucosylglucoside); Tectorigenin 4'-glucosyl(1->6)glucoside	Isoflavonoid O-glycosides	–	✓	✓	–	✓
Biochanin A 7-(6-malonylglucoside)	Isoflavonoid O-glycosides	✓	–	–	–	✓
Formononetin	4'-O-methylisoflavones	✓	–	✓	✓	✓
Genistein	Isoflavones	–	✓	✓	–	✓
Tectorigenin-4'-O-beta-D-glucoside; Tectorigenin 4'-glucoside	Isoflavonoid O-glycosides	–	–	✓	–	✓
2-(1,3-Benzodioxol-5-yl)-3,5,6,8-tetramethoxy-7-[(3-methyl-2-butenyl)oxy]-4H-1-benzopyran-4-one	8-O-methylated flavonoids	✓	–	✓	✓	–
Baicalein-7-O-glucuronide	Flavonoid 7-O-glucuronides	✓	✓	–	–	✓
Wogonin 7-glucuronide	Flavonoid 7-O-glucuronides	–	–	✓	–	✓
**IRIDOIDS / IRIDOID GLYCOSIDES**						
Asperulosidic acid	Iridoid O-glycosides	–	✓	✓	✓	✓
Asperuloside	Iridoid O-glycosides	✓	✓	–	–	✓
Genipin gentiobioside	Iridoid O-glycosides	✓	–	✓	–	–
**CARBOHYDRATES / GLYCOSYLATED COMPOUNDS**						
1-Kestose	Oligosaccharides	✓	✓	✓	✓	✓
4-O-Methyl-alpha-D-glucosyl-(1->2)-beta-D-xylosyl-(1->4)-D-xylose	Oligosaccharides	✓	✓	✓	✓	✓
D-(+)-Trehalose	O-glycosyl compounds	✓	✓	✓	✓	✓
D-Xylose	Pentoses	✓	✓	✓	✓	✓
Demethylisoalangiside	O-glycosyl compounds	✓	–	–	✓	–
Dihydrobotrydial	Oxanes	✓	✓	–	✓	✓
Glucosyl (E)-2,6-dimethyl-2,5-heptadienoate	Hexoses	✓	✓	✓	✓	✓
Phenethyl O-alpha-L-arabinopyranosyl-(1->6)-beta-D-glucopyranoside	O-glycosyl compounds	✓	✓	✓	✓	✓
Sucrose	Carbohydrates	✓	✓	✓	✓	✓
**CATECHINS / PROANTHOCYANIDINS**						
(-)-Epigallocatechin	Epigallocatechins	–	–	–	✓	✓

Phytochemical metabolite	Subclass	Leaf	Flower	Shoot	Root	Fruit
(-)-Epigallocatechin 3,3'-di-gallate	Catechin gallates	✓	✓	–	–	✓
(-)-Epigallocatechin 3-cinnamate	Epigallocatechins	✓	✓	✓	✓	✓
2-(3,4,5-trihydroxyphenyl)-3,4-dihydro-2H-1-benzopyran-3,4,5,7-tetrol	Epigallocatechins	✓	✓	✓	✓	–
Ellagic acid	Hydrolyzable tannins	✓	✓	✓	–	✓
Glucosyringic acid; Syringin 4-O-beta-glucoside	Hydrolyzable tannins	✓	✓	✓	✓	✓
Kandelin A1; Cinchonain-1a-(4beta->8)-catechin	Biflavonoids and polyflavonoids	✓	✓	–	–	✓
trans-3,3',4',5,5',7-Hexahydroxyflavanone	Epigallocatechins	✓	✓	✓	✓	✓
**COUMARINS**						
7-hydroxy-4-methylcoumarin	7-Hydroxycoumarins	✓	–	✓	✓	✓
Cichoriin	Coumarin glycosides	✓	✓	–	–	–
Daphnetin	7,8-Dihydroxycoumarins	–	✓	✓	–	✓
Scopoletin 7-glucoside	Coumarin glycosides	✓	✓	✓	–	✓
Umbelliferone	7-Hydroxycoumarins	✓	✓	✓	–	✓
**LIGNANS**						
(+)-7-epi-Syringaresinol 4'-glucoside	Lignan glycosides	✓	✓	✓	✓	✓
(-)-Isolariciresinol 4-O-beta-D-glucopyranoside	Lignan glycosides	✓	✓	✓	✓	✓
(7'R)-(+)-Lyoniresinol 9'-glucoside	Lignan glycosides	✓	✓	✓	✓	–
Liriodendrin	Lignan glycosides	✓	✓	✓	✓	–
Lyrioresinol A	Furanoid lignans	–	–	✓	✓	–
Piceaside G	Stilbene glycosides	✓	✓	✓	✓	✓
Secoisolariciresinol	Dibenzylbutanediol lignans	–	✓	✓	✓	✓
**TERPENES**						
(E)-Aldosecologanin	Terpene glycosides	✓	✓	✓	✓	–
(ent-2alpha,3beta,13Z)-3-Angeloyloxy-8(17),13-labdadiene-2,15,16,18-tetrol	Diterpenoids	✓	✓	✓	✓	✓
(1R*,2R*,3E,11R*,12S*)-2-O-acetyl-16-O-(3-hydroxy-3-methylglutaryl)-2,16,18-trihydroxy-dolabella-3,8(17)-dien-7-one	Diterpenoids	✓	✓	–	–	–
(S)-Abscisic acid	Abscisic acids and derivatives	✓	✓	✓	✓	✓
11-Dimethyl-3-[(acetyloxy)methyl]-7 2,6,11-dodecatriene-1,5,10-triol 1-acetate	Sesquiterpenoids	–	✓	✓	✓	✓
11alpha-Hydroxygedunin; (+)-11alpha-Hydroxygedunin	Limonoids	–	✓	–	–	✓
11beta,12alpha-Diacetoxysedrelone	Limonoids	–	–	✓	✓	–
13-Hydroxyabscisic acid	Abscisic acids and derivatives	–	✓	✓	–	–
15-Methoxyisodauc-3-ene-1beta,5alpha-diol	Germacrane sesquiterpenoids	✓	✓	✓	–	✓
2-acetoxy-7alpha-hydroxy-14-methoxy-5E-caryophyllene	Sesquiterpenoids	✓	✓	✓	✓	✓
28-Glucosyl-19(29)-dehydroursolic acid 3-arabinoside	Triterpene saponins	✓	–	–	✓	–
3-Hydroxy-beta-ionone	Sesquiterpenoids	✓	✓	✓	✓	✓
Azizic acid	Triterpenoids	–	–	✓	–	✓
Fraxinocarboside A	Terpene glycosides	–	–	✓	✓	–
Fusidic acid	Steroid esters	–	–	✓	✓	–
Geranylacetone	Acyclic monoterpenoids	✓	✓	✓	–	✓
Germacra-1(10),4,11(13)-trien-12-oic acid	Germacrane sesquiterpenoids	✓	✓	✓	✓	✓
Gitogenin	Triterpenoids	–	✓	✓	–	–
Hederagenin	Triterpenoids	–	–	✓	✓	✓
Juferin	Sesquiterpenoids	✓	✓	✓	✓	✓
Lupeol beta-phenylpropionate; Lupeol dihydrocinnamate	Triterpenoids	✓	✓	✓	–	–
Oleoside 11-methyl ester	Terpene glycosides	–	✓	✓	✓	✓

Phytochemical metabolite	Subclass	Leaf	Flower	Shoot	Root	Fruit
Visidic acid A; ent-15-Hydroxy-8(17),13E-labdadien-18-oic acid	Diterpenoids	–	–	✓	–	✓
**FATTY ACIDS AND LIPID DERIVATIVES**						
(S)-10,16-Dihydroxyhexadecanoic acid	Long-chain fatty acids	–	✓	✓	–	✓
(Z)-3-Hexenyl beta-D-glucopyranoside; (Z)-3-Hexen-1-ol-beta-D-glucopyranoside; (-)-(Z)-3-Hexenyl-beta-glucopyranoside	Fatty acyl glycosides of mono- and disaccharides	–	✓	✓	✓	–
16-Hydroxyhexadecanoic acid	Long-chain fatty acids	–	✓	✓	–	–
18-Hydroxy-9-octadecenoic acid	Long-chain fatty acids	✓	✓	✓	–	✓
20-Hydroxyeicosanoic acid	Long-chain fatty acids	–	–	✓	✓	–
22-Hydroxydocosanoic acid	Very-long-chain fatty acids	–	✓	✓	✓	✓
25-Hydroxypentacosanoic acid	Very-long-chain fatty acids	✓	✓	✓	✓	✓
26-Hydroxyhexacosanoic acid	Very-long-chain fatty acids	–	✓	✓	✓	–
3,7-Dimethyl-2,5-octadiene-1,7-diol	Fatty alcohol esters	✓	✓	✓	✓	✓
7-Epi-12-hydroxyjasmonic acid glucoside	Fatty acyl glycosides of mono- and disaccharides	✓	✓	✓	✓	✓
9,10-Epoxy-18-hydroxyoctadecanoic acid	Medium-chain fatty acids	✓	✓	✓	✓	✓
Docosanedioate(2-)	Very-long-chain fatty acids	–	✓	✓	✓	✓
Eicosenoic acid	Long-chain fatty acids	–	–	✓	–	✓
Fumaric acid	Dicarboxylic acids and derivatives	✓	✓	✓	✓	✓
Geranyl acetate	Fatty alcohol esters	✓	✓	✓	✓	✓
Hexadecanedioic acid	Long-chain fatty acids	✓	✓	✓	✓	✓
Linoleic acid	Linoleic acids and derivatives	✓	✓	✓	✓	✓
Octadec-9-ene-1,18-dioic acid	Long-chain fatty acids	–	✓	–	–	✓
Palmitic acid	Long-chain fatty acids	✓	✓	✓	✓	✓
Palmitoleic acid	Long-chain fatty acids	✓	–	✓	–	–
Panaxynol	Long-chain fatty alcohols	✓	–	✓	✓	✓
Pentadecanoic acid	Long-chain fatty acids	–	–	–	✓	✓
Petroselinic acid	Long-chain fatty acids	✓	✓	✓	✓	✓
Prenyl arabinosyl-(1->6)-glucoside	Fatty acyl glycosides of mono- and disaccharides	–	✓	–	–	✓
Punicic acid	Linoleic acids and derivatives	✓	✓	✓	✓	–
Ricinoleic acid	Long-chain fatty acids	✓	✓	✓	–	✓
Sebacate	Medium-chain fatty acids	–	✓	✓	✓	✓
Stearic acid	Long-chain fatty acids	✓	✓	✓	✓	✓
Suberic acid	Medium-chain fatty acids	✓	✓	✓	✓	✓
Succinic acid	Dicarboxylic acids and derivatives	✓	✓	✓	✓	✓

✓: detected; –: not detected.
